# Complementarity of assembly-first and mapping-first approaches for alternative splicing annotation and differential analysis from RNAseq data

**DOI:** 10.1038/s41598-018-21770-7

**Published:** 2018-03-09

**Authors:** Clara Benoit-Pilven, Camille Marchet, Emilie Chautard, Leandro Lima, Marie-Pierre Lambert, Gustavo Sacomoto, Amandine Rey, Audric Cologne, Sophie Terrone, Louis Dulaurier, Jean-Baptiste Claude, Cyril F. Bourgeois, Didier Auboeuf, Vincent Lacroix

**Affiliations:** 1 Université de Lyon, ENS de Lyon, Université Claude Bernard, CNRS UMR 5239, INSERM U1210, Laboratory of Biology and Modelling of the Cell, 46 Allée d’Italie Site Jacques Monod, F-69007 Lyon, France; 2 Université de Lyon, F-69000, Lyon; Université Lyon 1; CNRS, UMR5558, Laboratoire de Biométrie et Biologie Evolutive, F-69622, Villeurbanne, EPI ERABLE - Inria Grenoble, Rhône-Alpes, France; 3 IRISA Inria Rennes Bretagne Atlantique CNRS UMR 6074, Université Rennes 1, GenScale team, Rennes, 263 Avenue Général Leclerc, Rennes, France

## Abstract

Genome-wide analyses estimate that more than 90% of multi exonic human genes produce at least two transcripts through alternative splicing (AS). Various bioinformatics methods are available to analyze AS from RNAseq data. Most methods start by mapping the reads to an annotated reference genome, but some start by a *de novo* assembly of the reads. In this paper, we present a systematic comparison of a mapping-first approach (FaRLine) and an assembly-first approach (KisSplice). We applied these methods to two independent RNAseq datasets and found that the predictions of the two pipelines overlapped (70% of exon skipping events were common), but with noticeable differences. The assembly-first approach allowed to find more novel variants, including novel unannotated exons and splice sites. It also predicted AS in recently duplicated genes. The mapping-first approach allowed to find more lowly expressed splicing variants, and splice variants overlapping repeats. This work demonstrates that annotating AS with a single approach leads to missing out a large number of candidates, many of which are differentially regulated across conditions and can be validated experimentally. We therefore advocate for the combined use of both mapping-first and assembly-first approaches for the annotation and differential analysis of AS from RNAseq datasets.

## Introduction

In the last 10 years, the prevalence of alternative splicing has been completely re-evaluated. Recent reports claim that more than 90% of multi-exon genes produce at least two splicing variants^1,2^. The depth at which transcriptomes can be sampled with next generation sequencing techniques opens the possibility not only to annotate splicing variants in various conditions, but also to detect which transcripts are differentially spliced across pathological and physiological conditions.

This growing interest in splicing both as a fundamental process and because of its implication in pathologies^3–5^ has been accompanied by an increasing number of methods aiming at analyzing RNAseq datasets^6–8^. The ultimate goal of these methods is to identify and quantify full-length transcripts from short sequencing reads. This task is particularly challenging and recent benchmarks show that all methods still make a lot of mistakes^9^. The difficulty of reconstructing full-length transcripts (isoform-centric approaches) also prompted a number of authors to focus on identifying exons that are differentially included within transcripts (exon-centric approaches)^10–13^.

Whether they are exon- or isoform-centric, methods to study splicing from RNAseq data can further be divided in two main categories^14^. The mapping-first approaches first map the reads to the reference genome and the mapped reads are then assembled into exons and eventually transcripts. In contrast, assembly-first approaches first assemble the reads based on their overlaps. The assembled sequences (corresponding to sets of exons) are then aligned to the reference genome.

Mapping-first approaches have been the most used so far, essentially because they were the first to be developed and because they initially required less computational resources. *De novo* assembly methods were also thought to be restricted to non-model species, where no (good) reference genome is available, and they seemed to be inadequate when an annotated reference genome is available.

Recent progress in *de novo* transcriptome assembly is clearly changing this view, and the argument of the heavier computational burden does not hold anymore.

The application of *de novo* assembly to human RNAseq datasets however still remains rare, although some studies have already shown its potential to detect novel biologically relevant splicing variants^15,16^.

The generalization of *de novo* assembly approaches for studying splicing in human seems to be mostly impeded by the lack of a clear evaluation of its potential interest in comparison to more traditional mapping-based approaches.

This is the gap we aim at filling with the work presented here.

To achieve this goal, we performed a systematic evaluation of an assembly-first and a mapping-first approach on two RNAseq datasets.

As a first step, we compared pipelines that we developed in parallel, namely KisSplice and FaRLine, because we could easily control their parameters. Any difference between the predictions that is solely due to a parameter setting could be fixed easily, which enabled us to obtain a precise understanding of the irreducible differences between the two approaches.

In a second step, we confirmed the generality of our findings by benchmarking our methods against Cufflinks^6^, MISO^11^ and Trinity^17^, which are widely used pipelines.

A significant part of our work has been to manually dissect a number of cases found by only one of the two methods. This enabled us to go beyond a simple qualitative description and provide the community with a precise understanding of which cases are overlooked by each type of method, and where new methods are needed.

All the software and step-by-step protocols presented in this work are freely available at http://kissplice.prabi.fr/pipeline_ks_farline. This should facilitate the reproducibility of our work, and applications to other datasets.

From a general point of view, the combination of approaches we propose should enable to improve splicing-related transcriptomic analyses in physiological and pathological situations.

## Results

### KisSplice and FaRLine

**Figure 1:**
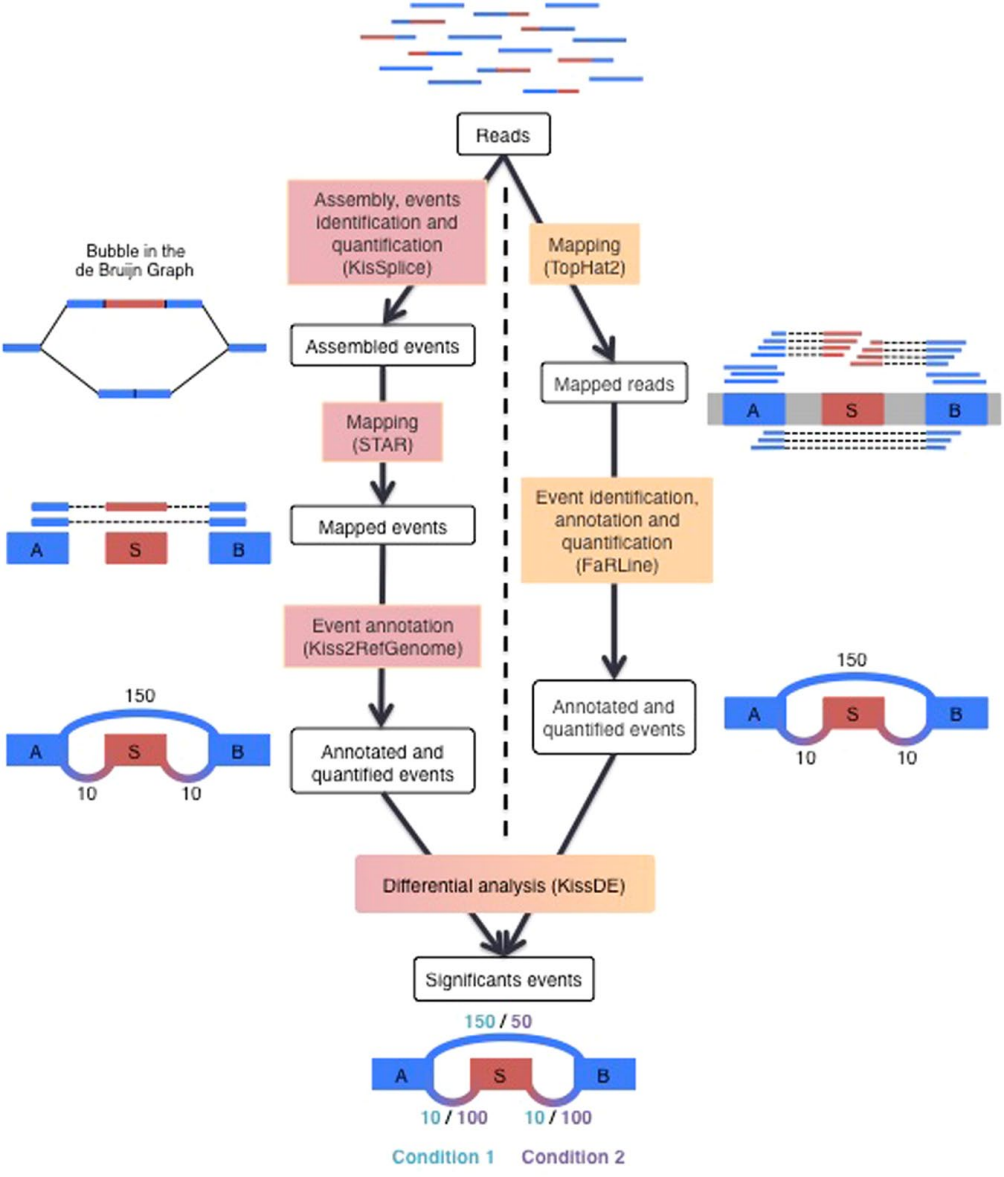
The two pipelines compared in this study: KisSplice and FaRLine. The first step of KisSplice is to assemble the reads and extract the splicing events. These events are then mapped back to the reference genome and classified by event type. The annotated and quantified events are then used for the differential analysis between the biological conditions. In contrast, the first step of FaRLine is to map the reads on the reference genome. From this mapping, annotated and quantified events are extracted. Finally, the differential analysis is done with the same method as in the KisSplice pipeline.

Figure 1 presents schematically the two pipelines that we developed and compared. A detailed description of each step is given in the Methods section. In the assembly-first approach, a De Bruijn graph is built from the reads. Alternative splicing events, which correspond to bubbles in this graph are enumerated and quantified by KisSplice. Each path is then mapped on the reference genome using STAR and the event is annotated by KisSplice2RefGenome, using the EnsEMBL r75 annotations as an evidence. Importantly, exons not present in the annotations can be identified by this approach. In the mapping-first approach, reads are aligned to the reference genome using TopHat2. Mapped reads are then analyzed by FaRLine, using the EnsEMBL r75 annotations as a guide.

We also tested STAR instead of TopHat2 for the mapping-first pipeline, and found that our main results were essentially unchanged (see Methods).

Quantification of splicing variation is performed similarly in the two pipelines. Only junction reads are considered. Exonic reads are not considered, for reasons exposed in Methods. For the inclusion isoform, there are two junctions to consider. We calculate the mean of the counts of these two junctions.

The differential analysis is performed by a common method for the two approaches: kissDE, which tests if the relative abundance of the inclusion isoform has changed significantly across conditions.

Overall, we developed and adapted jointly these two pipelines in order to minimize the discrepancies that could complicate the comparison.

### The majority of frequent isoforms are identified by both approaches

**Figure 2:**
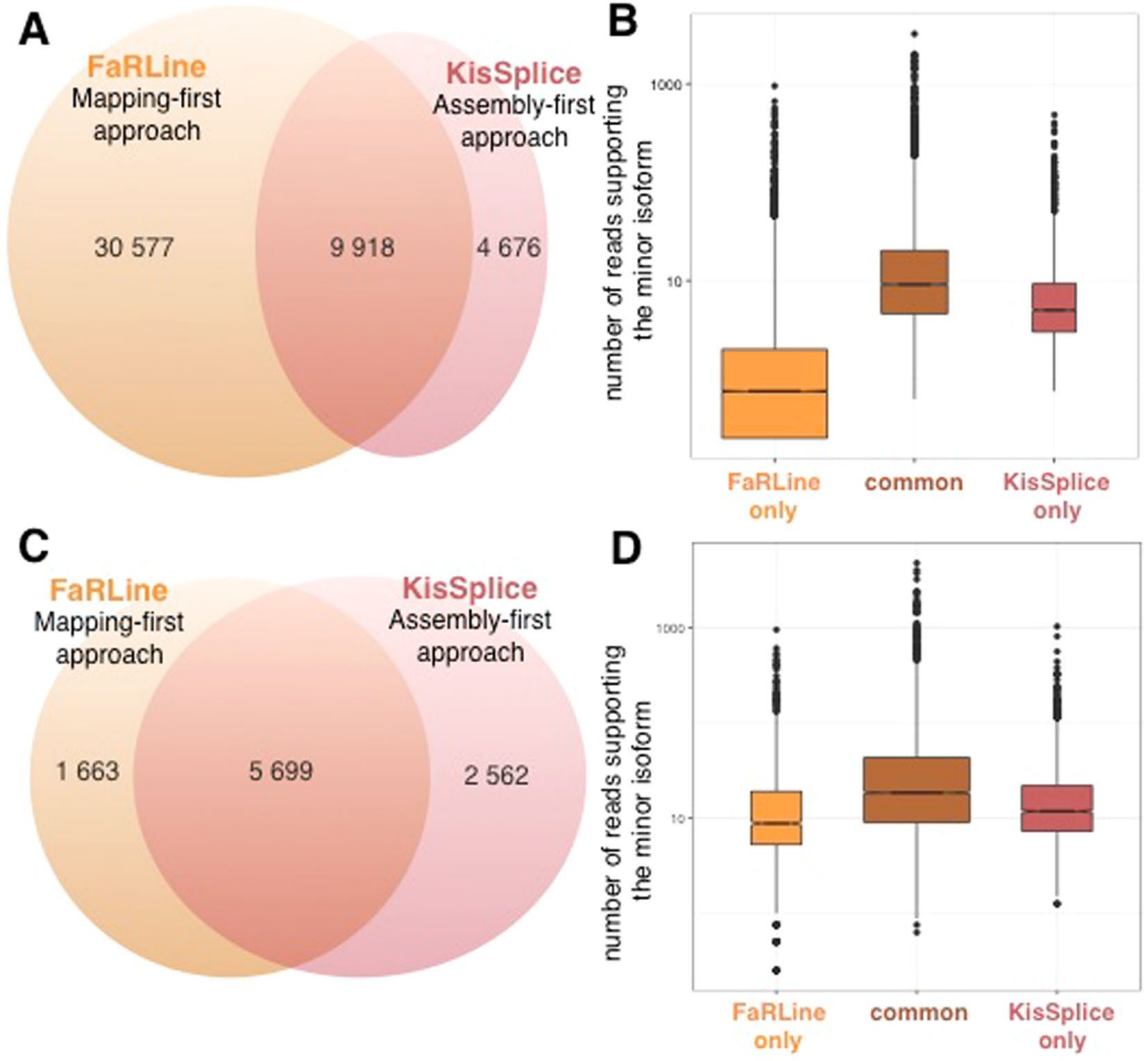
Comparison of the ASE identified by the assembly-first and mapping-first pipelines. (**A**) Venn diagram of ASEs identified by the two pipelines. FaRLine detected many more events than KisSplice. 68% of ASE found by KisSplice were also found by FaRLine and 24% of ASE detected by FaRLine were also found by KisSplice. (**B**) Boxplot of the expression of the minor isoform in the 3 categories defined in the Venn diagram of panel A: ASE identified only by FaRLine, ASE identified by both pipelines and ASE identified only by KisSplice. The number of reads supporting the minor isoform of the ASE identified by FaRLine is overall much lower. Many isoforms are supported by less than 5 reads. (**C**) Venn diagram of ASEs identified by the two pipelines after filtering out the poorly expressed isoforms (less than 5 reads, or less than 10% of the number of reads supporting both isoforms). The common events represent a larger proportion than before filtering: 77% of the ASE identified by FaRLine and 69% of the ASE identified by KisSplice. (**D**) Boxplot of the expression of the minor isoform in the 3 categories defined in the Venn diagram of panel C: ASE identified only by FaRLine, ASE identified by both pipelines and ASE identified only by KisSplice. The distribution of the number of reads supporting the minor isoform is similar for the 3 categories with highly expressed variants in each category.

Applying KisSplice and FaRLine to the same RNAseq datasets generated by the ENCODE consortium (SK-N-SH cell lines treated or not with retinoic acid), we noticed that 68% of the alternatively skipped exons (ASE) identified by KisSplice were also identified by FaRLine and that 24% of ASEs identified by FaRLine were also identified by KisSplice (Fig. 2A). This observation highlights that the mapping-first approach predicts a much larger number of events. This difference in sensitivity is due to the fact that while mapping-first approaches require that each exon junction is covered by at least one read, assembly-first approaches require overlapping reads across the entire skipped exon. Therefore, it can be anticipated that low abundant isoforms, that are covered by few reads, will be reported by mapping, but not by the assembly-first approach. Supporting this prediction, we observed that for ASEs reported only by FaRLine, the number of reads supporting the minor isoform is much lower than in the other categories (Fig. 2 B). The same results were obtained using another RNAseq dataset representing MCF-7 cells expressing or not the DDX5 and DDX17 splicing factors (Supplementary Figure S1).

Having clarified that rare variants are better handled by the mapping-first approach, we decided to filter them out, in order to analyse other differences between the two approaches. Experimental validations by RT-PCR that we performed on rare variants stratified by read support enabled us to clarify that both an absolute and a relative cutoff on the number of reads are required to discriminate variants which can be validated from those which cannot. Indeed, out of the 48 tested cases, we were able to validate 41 (Supplementary Figure S9). The non validated cases indeed corresponded to cases supported by fewer reads. However, what really departed them from the validated cases was their lower relative abundance (Supplementary Figure S10, Supplementary Table 1). In the remaining of our work, we chose to use both criteria and we filtered variants supported by less than 5 reads, and less than 10% compared to the major isoform.

As expected, the proportion of candidates reported simultaneously by both methods increased significantly. Approximately 70% of predicted skipped exons were indeed found by both approaches after filtering lowly expressed isoforms. (Fig. 2C, Supplementary Figure S1C).

Furthermore, the estimation of their inclusion rates was consistent across the two approaches (*R*^2^ > 0.9)).

Beyond the overall concordance of the two approaches in detecting common splicing events, a number of candidates remained reported by only one approach. Since many of them have a highly-expressed minor isoform (supported by more than 100 reads) (Fig. 2D, Supplementary S1D), the failure of one approach to detect them is likely not due to a lack of coverage.

**Figure 3:**
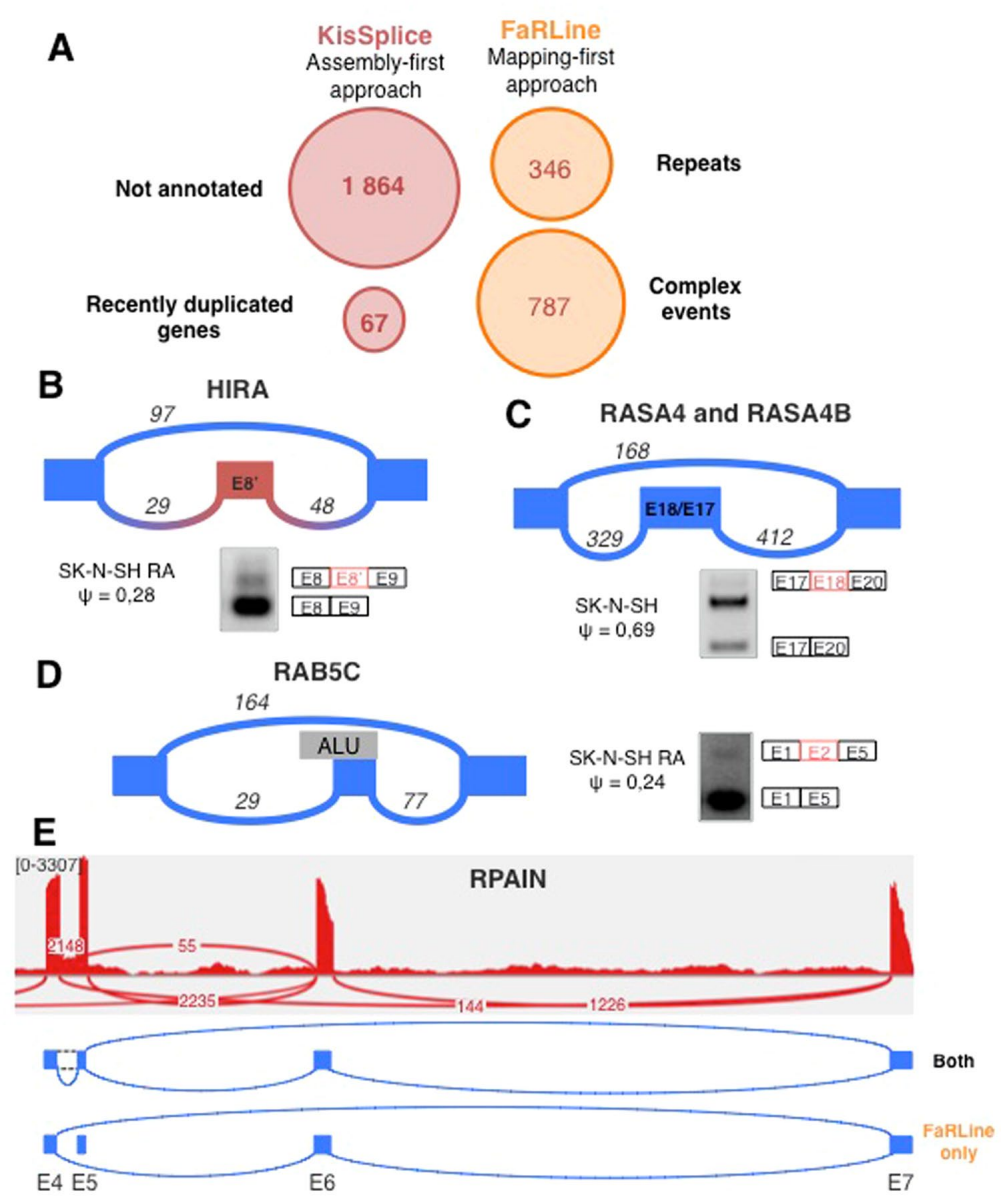
(**A**) Main categories explaining why some exons are detected by only one method. (**B**) The exon in intron 8 of the *HIRA* gene is an example of an exon not annotated in EnsEMBL r75. This event was identified by KisSplice but not by FaRLine. (**C**) *RASA4* and *RASA4B* are 2 paralog genes. KisSplice detected 2 isoforms that could be produced by these 2 genes. FaRLine did not detect any event in either of these genes. The exon skipped is exon 18 in *RASA4* (corresponding to exon 17 in *RASA4B*). The third band on the RT-PCR is the inclusion of another exon in the intron 18 of *RASA4*. (**D**) Exon 2 of the *RAB5C* gene is an example of exon skipping overlapping an Alu element identified only by FaRLine. The events in panel B to C were validated by RT-PCR. (**E**) The *RPAIN* gene contains a complex event with a lowly expressed isoform. This weakly expressed isoform was not identified by KisSplice, while the other isoforms were identified by both approaches.

For events only found by one approach, we patiently dissected the reasons why they could have been missed out by the other approach. This enabled us to define 4 main categories which cover 70% of the cases (Fig. 3A) The remaining 30% of cases did not fit into clearly defined biological categories. We however classified them using methodological criteria. The full list of categories is presented in Supplementary Table 2. For each of the 4 main categories, we selected cases to validate experimentally. All 34 RT-PCR validations were successful and are presented in Supplementary Figure S11 confirming that these events are not false positives.

### Some isoforms are systematically missed by one approach

The first category corresponds to cases that were missed out by the mapping-first approach and corresponds to alternative splicing events involving novel exons or novel combinations of existing exons.

There are two reasons to explain why the mapping-first approach does not detect these events. First the mapper may fail to map the reads, or map them to an incorrect location, as junction discovery using short reads is a challenging task. Second, even in the case where the mapper succeeds, FaRLine may fail to report the event because it relies on annotations. Among these 1864 cases, we distinguished 3 sub-categories of errors due to the annotation. Either the exon is unannotated (30%), one of its flanking exon is unannotated (13%) or both exons are annotated but no transcript combining them was annotated (57%).

The assembly-first approach, KisSplice, does not consider annotations, and an interesting resulting advantage is that novel junctions have the same chance to be assembled as known junctions. Mapping assembled novel junctions to the genome is indeed less challenging than read mapping because the assembled sequences are longer.

More importantly, the ability of KisSplice to identify novel splicing events comes from the fact that it introduces known annotations as late as possible in its pipeline (see Methods). Annotations are used as an evidence, not as a filter. AS events involving novel splice sites are clearly identified as such, and can be specifically tested and experimentally validated. More than 99% of the novel splice sites were canonical splice sites (GT-AG).

As an example, the *HIRA* gene contains a novel exon, whose inclusion is supported by at least 20 reads on each junction (Fig. 3B, Supplementary Figure S8A). This case was overseen by the mapping-first approach, FaRLine. The panel B of the Supplementary Figure S8 shows an example of an ASE not reported by FaRLine because the included exon was not present in the transcripts.

The second category of splicing events identified by only one approach corresponds to recent gene duplications. Untangling the relation between alternative splicing and gene duplication is a difficult topic, subject to debate^18,19^. It is indeed difficult to assess the amount of alternative splicing that occurs within paralogous genes. With the mapping-first approach, the reads stemming from recent paralogs are classified as multi-mapping reads. FaRLine, like the vast majority of mapping-first pipelines, discards these reads for further analysis, as their precise location cannot be clearly established. This results in silently underestimating alternative splicing in recent paralog genes. Note that setting the mapper to keep multi-mapping reads in the analysis leads to overestimating alternative splicing, as all members of the family will be predicted as alternatively spliced. In opposition, *de novo* assembly can faithfully state that a family of recent paralogs collectively produce two isoforms that vary in their sequence. However, whether the two isoforms are produced from the same locus or from different loci remains undetermined. KisSplice detects these cases of putative AS in paralog genes. Figure 3C illustrates the case with genes *RASA4* and *RASA4B*. Exon 18 in *RASA4* (denoted as exon 17 in *RASA4B*) was detected to be skipped. The exclusion isoform is supported by 160 reads, while the inclusion isoform is supported by 400 reads. The mapping-first approach did not detect either of these isoforms at all. Another example from this category is presented in Supplementary Figure S2C.

The third category of splicing events identified by only one approach corresponds to cases that are missed out by the assembly-first approach. Out of the 1663 cases belonging to this category, a large fraction (21%) corresponds to cases where the skipped exon overlaps a repeat, notably Alu elements. Alu are transposable elements present in a very large number of copies in the human genome^20^. Most of these copies are located in introns and a number of them have been exonised^21,22^. The reason why the mapping-first approach is able to identify these cases is because even though the reads partially map to repeated sequences, the boundaries of these exons are unique and annotated. Hence the mapper, if set properly, can map these reads to unique annotated exon junctions and is not confused by multiple mappings. Importantly, if the annotations are not provided to the mapper, it will be confused by multiple mappings and will not be able to map the read to the correct location (Supplementary Figure S7). Figure 3D and Supplementary Figure S2D represent two RT-PCR validated Alu-derived exons identified by the mapping-first approach. The assembly-based approach fails to detect most of these events. The reason is that, although they do form bubbles in the DBG generated by the reads, these bubbles are highly branching (supplementary figure http://kissplice.prabi.fr/sknsh/graph_RAB5C_distance_3.html23). Enumerating branching bubbles is computationally very challenging, and may take a prohibitive amount of time. In practice, we restrict our search to the enumeration of bubbles with at most 5 branches (Supplementary Figure S12A).

The fourth category of splicing events identified by only one approach corresponds to cases where more than two splicing isoforms locally coexist, and one of them is poorly expressed compared to the others. The *RPAIN* gene is a good illustration of such cases (Fig. 3E), as exons 5 and 6 of *RPAIN* may be skipped and the intron between exons 4 and 5 may be retained. While both methods successfully reported the skipping of exon 6, with exons 5 and 7 as flanking, FaRLine additionally reported the skipping of the same exon, but with exons 4 and 7 as flanking exons. The reason why KisSplice did not report this case is because the junction between exons 4 and 6 is relatively weakly supported. More specifically, this junction is supported by only 55 reads, which accounts for less than 2% of the total number of reads branching out from exon 4. Transcriptome assemblers, like KisSplice, usually interpret such relatively weakly supported junctions as sequencing errors or spurious junctions in highly-expressed genes, therefore disregarding them in the assembly phase (see Supplementary Methods). Supplementary Figure S2E shows another example of a complex event not correctly handled by KisSplice because there were locally more than 5 branches.

### Comparison of the approaches after differential analysis

Beyond the tasks of identifying exon skipping events, a natural question which arises when two conditions are compared is to assess if the exon inclusion rate significantly changed across conditions.

**Figure 4:**
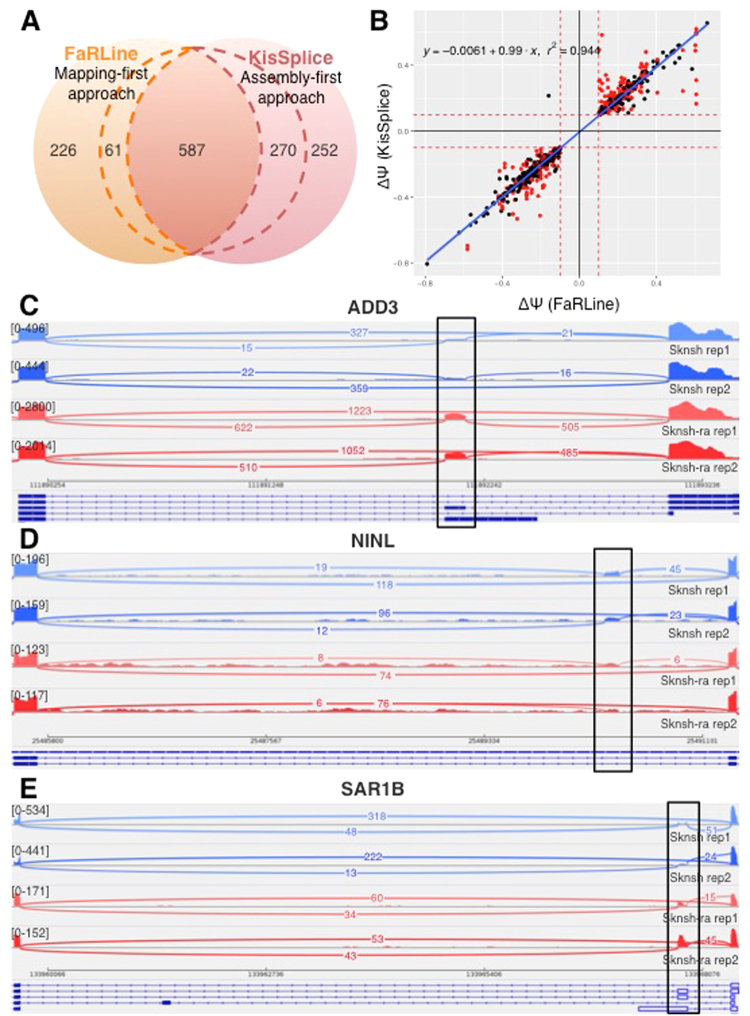
(**A**) Condition-specific variants identified by FaRLine, KisSplice or both methods. Within dashed lines are events identified by both approaches but detected as condition-specific by only one approach. (**B**) DeltaPSI as estimated by KisSplice and FaRLine, for events identified by both methods. The red dots represent complex events for which KisSplice found at least 2 ‘bubbles’. (**C**) Exon 22 of the *ADD3* gene is an example of regulated ASE identified by both approaches. (**D**) A new exon in intron 5 of *NINL* gene is identified only by KisSplice. The inclusion of this exon is differentially regulated between the 2 experimental conditions. (**E**) Because exon 3 of the *SAR1B* gene is an exonised Alu element, only FaRLine identified this event. Moreover this exon is significantly more included in the treated cells (SK-N-SH RA) compared to the control cells.

In order to test this, we took advantage of the availability of replicates for both the SK-N-SH cell line and the same cell line treated with retinoic acid. For each detected event, we tested with kissDE^24^, whether we could detect a significant association between one isoform and one condition. Focusing on those condition-specific events, we again partitioned them in events reported by both methods, by FaRLine only and by KisSplice only. As shown in Fig. 4, the majority of condition-specific events were detected by both approaches. This is the case for instance of exon 22 of gene *ADD3* which is clearly more included upon retinoic acid treatment (Fig. 4C), with a DeltaPSI of 27%. The estimation of the DeltaPSI is overall very similar across the two approaches (Fig. 4B) with a correlation of 0.94. The outliers essentially correspond to ASE with several alternative donor/acceptor sites. KisSplice considers these events as different exons while FaRLine considers them as an unique exon, and sums up all the incoming (resp. outgoing) junction counts. Hence, the read counts will differ. Supplementary Figure S8D gives an example.

When focusing on condition-specific events, the proportion of events predicted by only one method increased, for two main reasons. First, some ASE annotated by both approaches were predicted to be differentially included only by one method. This is again due to differences in the quantification of the inclusion rate, especially for ASE with multiple 5′ and 3′ splice sites. Second, some of the exons that were missed out by one method at the identification step happened to be condition specific. This is the case of an exon in *NINL* intron 5 (Fig. 4D), only identified by KisSplice because it was not annotated. This is also the case of *SAR1B* exon 3 (Fig. 4E), only identified by FaRLine because it overlaps with an Alu element. The analysis of the MCF-7 RNAseq dataset gave very similar results (Supplementary Figure S3).

The observation that many of the AS events that were annotated only by one method are differentially regulated across conditions confirms that these AS events should not be discarded from the analysis. Focusing only on AS events annotated by one approach may lead to miss splicing events which are central in the biological context.

### Overlap with other methods

**Figure 5:**
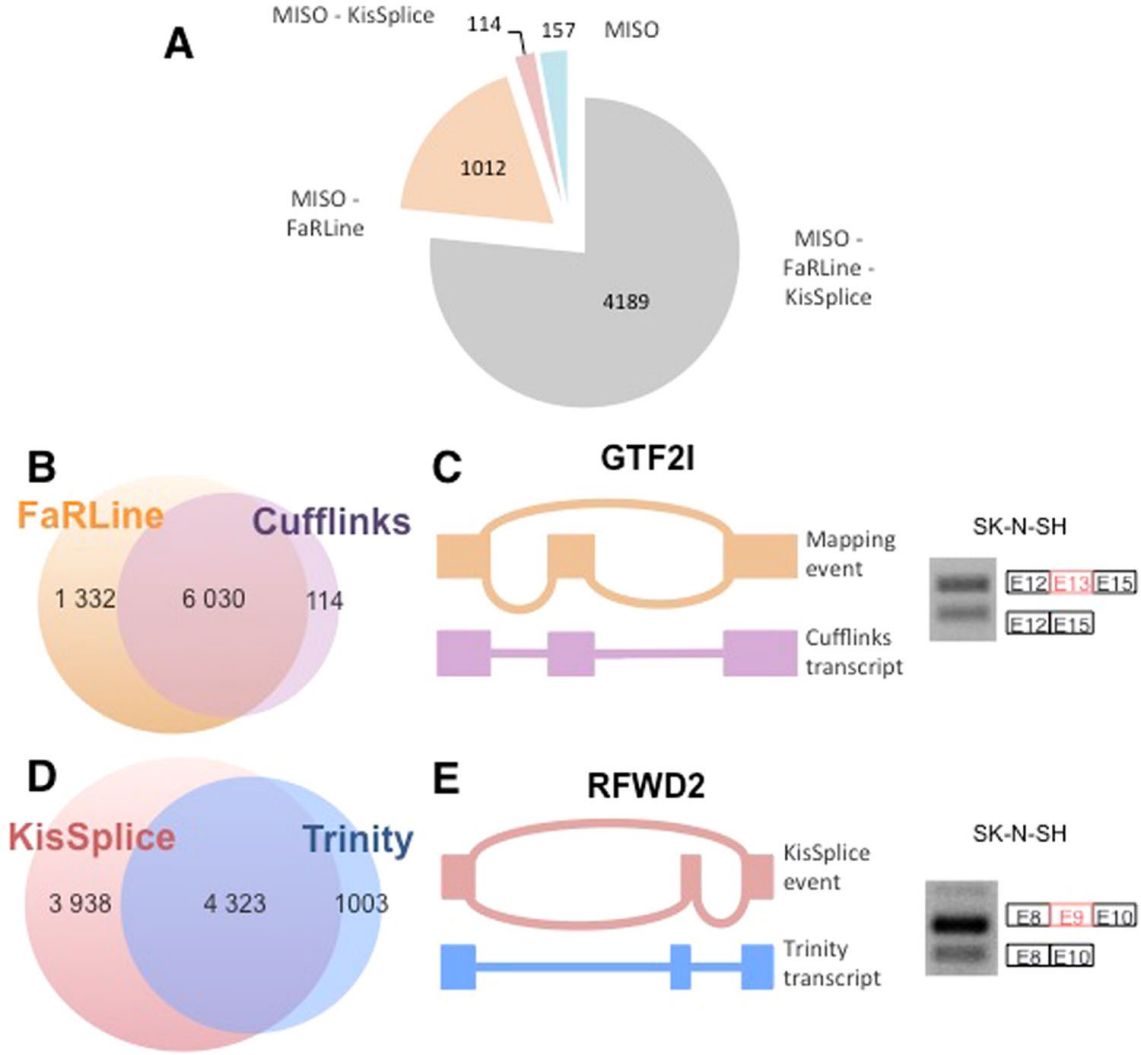
(**A**) 77% of ASE identifed by MISO are also annotated by FaRLine and KisSplice. 18% of MISO’s ASE are also annotated by FaRLine while only 2% of MISO’s ASE are also annotated by KisSplice. Finally, only 3% of these ASEs are only annotated by MISO. (**B**) Most of the events annotated by Cufflinks are identified by FaRLine. (**C**) *GTF2I* exon 13 is an example of an ASE annotated by FaRLine but not by Cufflinks. Indeed, Cufflinks only identified the isoform corresponding to the exon inclusion. (**D**) Most of the events annotated by Trinity are also annotated by KisSplice. But half of the ASE annotated by KisSplice are not annotated by the global assembler Trinity. (**E**) KisSplice annotates an ASE in the *RFWD2* gene, while Trinity only identified the isoform corresponding to the exon inclusion. The events in panels C and E have been validated by RT-PCR.

In a first step, we picked FaRLine and KisSplice as examples of a mapping-first and an assembly-first approach respectively. Clearly, there are other published methods in both categories. MISO is probably the most widely used to annotate AS events. We therefore ran it on the same datasets to check how its predictions overlapped with ours. As shown in Fig. 5A (SK-N-SH dataset), 77% of predictions made by MISO were common to both FaRLine and KisSplice, 18% were only common with FaRLine, 2% were only common to KisSplice and the remaining 3% were specific to MISO. The overlap between the different methods was very similar when the MCF-7 RNAseq dataset was used (Supplementary Figure S4A). Overall, almost all candidates predicted by MISO were also predicted by FaRLine. This large overlap with FaRLine was expected, because both are mapping-first approaches. This also shows that the differences between mapping- and assembly-first approaches reported above are not limited to one mapping-first approach.

Besides exon-centric approaches, which aim at finding the differentially spliced exons, there is also a number of published methods which are isoform-centric and have the more ambitious goal to reconstruct full-length transcripts at the expense of underestimating alternative splicing.

The most widely used mapping-first and isoform-centric approach is Cufflinks^6^ that we compared to FaRLine using the same dataset. As shown in Fig. 5B (and Supplementary Figure S4B), we found that the vast majority of ASE were predicted by both approaches.

Finally, we compared KisSplice to one of the most widely used de-novo transcriptome assembler, Trinity^17^. As shown in Fig. 5D (and Supplementary Figure S4D), most ASE found by Trinity were also found by KisSplice. However, KisSplice was significantly more sensitive. The goal of Trinity is to assemble the major isoforms for each gene, it therefore largely under-estimates alternative splicing, especially inclusion/exclusion of short sequences.

For completeness sake, we also provide an all-vs-all comparison (Supplementary Figure S5). An interactive version of this Figure is available at http://kissplice.prabi.fr/pipeline_ks_farline/. The list of events found by any used method can be retrieved from this interactive figure and analysed in IGV, to reproduce the sashimi plots of the paper. The general conclusions from these comparisons is that there is a clear distinction between mapping-first and assembly-first approaches, and between exon-centric and isoform-centric approaches, the latter being less sensitive.

## Discussion

*De novo* assembly is usually applied to non-model species where no (good) reference genome is available. We show here that even when an annotated reference genome is available, using assembly offers a number of advantages. We named this approach “assembly-first” because it does use a reference genome, but as late as possible in the process, in order to minimize the *a priori* on which exons should be identified.

Using this strategy, we identified novel alternatively skipped exons, which were not identified by traditional read mapping approaches (Fig. 3 and Supplementary Figure S2). While it is believed that the human genome is fully annotated, it is important to underline that we have not yet established a final map of the parts of the genome that can be expressed. It can be anticipated that sequencing of single-cells from different parts of the body will lead to the discovery of a huge diversity of transcripts and that a substantial number of new exons will be discovered. An example is the case of unannotated skipped exons which overlap with repeat elements. We cannot exclude that this category is currently largely under-annotated.

We also showed that assembly-first approach has the ability to detect splicing variants within recently duplicated genes (Fig. 3 and Supplementary Figure S2). This is because mapping approaches discard reads which map to multiple genomic locations. Identification of such splicing variants produced from different genomic regions sharing sequence similarities (e.g. paralog genes, pseudogenes) is however very important, since splicing variants generated from paralogous genes but also from pseudogenes may have different biological functions^25^.

Conversely, we showed that some ASE were detected only by the mapping-first approach. As shown in Fig. 2 (and Supplementary Figure S1), we observed that the mapping-first approach has a better ability to detect lowly-expressed splicing variants. Although such lowly-expressed splicing variants are often considered as “noise” or biologically non relevant, caution must be taken with such assumptions for several reasons. First, mRNA expression level is not necessarily correlated with protein expression level. Second, as observed from single-cell transcriptome analyses, some mRNAs can be expressed in few cells, within a cell population (e.g. they are expressed at a specific cell cycle step) and may therefore appear to be expressed at a low level in total RNAs extracted from a mixed cell population^26^. Therefore, computational analysis should not systematically discard lowly-expressed splicing variants and filtering these events should depend on the biological questions to be addressed.

We also observed that the mapping-first approach better detects exons corresponding to annotated-repeat elements (Fig. 3 and Supplementary Figure S2). While it has been assumed for a long time that repeat elements are “junk”, increasing evidences support important biological functions for such elements. For example, repeat elements like Alu can evolve as exons and the presence of Alu exons in transcripts has been shown to play important regulatory functions^22,27^.

When two methods give non-overlapping predictions, the temptation could be to focus on exons found by both approaches and to discard the others. We argue that this is not the best option, because approach-specific cases can be validated experimentally, and also because many of them correspond to regulated events, i.e. the inclusion isoform is significantly up or down regulated depending on the experimental condition.

In conclusion, combining mapping- and assembly-first approaches allows to detect a larger diversity of splicing variants. This is very important towards the in depth characterization of cellular transcriptome although other approaches are further required to analyze their biological functions.

From a computational perspective, a number of challenges are still ahead. The co-development of two approaches enabled us to narrow down the list of difficult instances not properly dealt with by at least one approach, but we cannot exclude that some categories are still missed out by both approaches. The categories of challenging cases that we defined in Fig. 3: lowly-expressed variants, exonised Alu, complex splicing variants, paralogs have been overlooked up to now. Possibly because they are much harder to detect, they have been assumed to play a minor role in transcriptomes, but more recent studies however argues the opposite.

For exonised ALUs, paralog genes and genes with complex splicing patterns, the possibility to sequence longer reads with third generation techniques^28,29^ should prove very helpful. The number of reads obtained with these techniques is however currently much lower than with Illumina, thereby preventing their widespread use for differential splicing, for which the sequencing depth, and not so much the length of the reads, is the critical parameter which conditions the statistical power of the tests. In the coming years, methods combining second and third generation sequencing should enable to obtain significant advances in RNA splicing.

## Material and Methods

### FaRLine and KisSplice

Figure 1 shows the two pipelines that we are comparing. While STAR and TopHat are third-party softwares, we developed the other methods ourselves. KisSplice was first introduced in Sacomoto *et al*.^13^. The novelty here is that its usage is now possible in the case where a reference genome is available, which required specific methodological developments implemented in the newly released KisSplice2RefGenome software. kissDE was first introduced in Lopez-Maestre *et al*.^24^ in the context of SNPs for non-model species. We present here its extension for alternative splicing. FaRLine is a new mapping-first pipeline, that we introduce in this paper. It is the RNAseq pipeline associated to the FasterDB database^30^ and was already successfully applied to the analysis of the effect of metformin treatment on myotonic dystrophy type I (DM1) with a validation rate of 95%^31^. Specifically, 20 cases of ASE regulated by the metformin treatment were tested, and 19 were validated. In this paper, we provide additional validations of FaRLine with similar validation rates (36 out of 38), Supplementary Figure S19.

For the sake of self-containment, we explain all methods here.

#### KisSplice

KisSplice is a local transcriptome assembler. As most short reads transcriptome assemblers^8,17,32^, it relies on a De Bruijn graph (DBG). Its originality lies in the fact that it does not try to assemble full-length transcripts. Instead, it assembles the parts of the transcripts where there is a variation in the exon content. By aiming at a simpler goal, it can afford to be more exhaustive and identify more splicing events. The key concept on which KisSplice is built is that variations in the nucleotide content of the transcripts will correspond to specific patterns in the DBG called bubbles (Supplementary Figure S13). KisSplice’s main algorithmic step therefore consists in enumerating all the bubbles in the graph built from the reads. Examples of bubbles in the DBG and explanation of the parameters used to filter out sequencing errors and repeat-induced bubbles are given in Supplementary Methods.

#### Annotating the events with KisSplice2RefGenome

KisSplice outputs bubbles in the form of a pair of fasta sequences. Clearly, such information is insufficient to analyse alternative splicing for model species. KisSplice2RefGenome enables to provide for each bubble: the gene name, the AS event type, the genomic coordinates and the list of splice sites used (novel or annotated).

Bubbles found by KisSplice are mapped to the reference genome using STAR, with its default settings, which means that in the case of multi-mappings, STAR reports all equally best matches. The mapping results are then analysed by KisSplice2RefGenome. Bubbles are classified in sub-types depending on the number of blocks obtained when mapping each path of the bubble to the genome (Supplementary Figure S14). For exon skipping, the longer path of the bubble corresponds to 3 blocks, while the lower path corresponds to 2 blocks. The splice sites are located and compared to the annotations. Events with novel splice sites are reported explicitly as such in the output of the program.

In the case where the bubble corresponds to a genomic insertion or deletion, it exhibits a specific pattern in terms of block numbers (one block for one path and two blocks for the other) and is reported separately.

The criterion of the number of blocks is discriminative in most cases. However, there is a possible confusion between intron retentions and genomic deletions, since in both cases, the longer path will map into one block and the lower path in two blocks. In this case, we also use the distance between the blocks, and introduce a user-defined threshold, which we set to 50nt, below which the bubble is classified as a genomic deletion, and above which it is classified as an intron retention.

In the special case where the exon flanking the AS event is very short (less than k nt), the number of blocks is increased for both paths, but the difference of number of blocks remains unchanged.

In the special case where there is a genomic polymorphism located less than k nt apart from the AS event, KisSplice will report several bubbles (possibly all combinations of genomic and transcriptomic variants). This redundancy is removed in KisSplice2RefGenome where the primary focus in on splicing.

In the case where the bubble maps to two locations on the genome, a distinction is made between the case of exact repeats where both paths map to both locations and inexact repeats where each path maps to a distinct location (Supplementary Figure S12B). The cases of exact repeats correspond to recent gene duplications.

#### FaRLine

**FasterDB EnsEMBL r75 annotation:** FasterDB RNAseq Pipeline, FaRLine, uses the FasterDB-based EnsEMBL r75 annotation database. FasterDB is a database containing all annotated human splicing variants^30^.

Each transcripts present in the FasterDB, is composed of a succession of exons, that we call transcript exons (represented in blue in Supplementary Figure S15). The genomic exons (represented in red in Supplementary Figure S15) are defined by projecting the transcript exons. First, the transcript exons are grouped by position. Then each group of exons defines a projected exon with the following rules:

- The start is the leftmost start of the non-first-exon of the group.
- The end is the rightmost end of the non-last-exon of the group that ends before the start of the next group of exons.

When the most frequent event annotated in the transcripts is an intron retention, the projected genomic exon is defined as a combination of the two exons flanking the retained intron. In Supplementary Figure S15, the exons 5 and 6 and the intron 5 are considered as one unique exon. As events included within one exon are not tested, this results in some events being missed.

**Mapping:** The first step of FaRLine is to map the reads to a reference genome. This step is done using Tophat-2.0.11^6^. tophat–min-intron-length 30–max-intron-length 1200000\-p 8 [–solexa1.3-quals for Sknsh_rep1 and Sknsh_rep2]\–transcriptome-index

A transcriptome index has been built by TopHat using EnsEMBL r75 annotations in gtf format. When a transcriptome index is used, the mapping steps are modified: instead of aligning first to the genome, which is the default behavior, TopHat uses Bowtie to align the reads to the transcript sequences first, then align the remaining unmapped reads to the genome. Minimal and maximal intron lengths have been modified (default 70 and 500000) to maximize the number of junctions detected, according to the statistics provided by FasterDB EnsEMBL r75 annotations.

The resulting alignment files have been filtered using samtools 0.1.19^33^.

Samtools view -F 260 -f 1 -q 10 -b

With this step, only the primary alignments are kept. The minimum read alignment quality was set up so that multi-mapping reads were removed from the alignment file.

**Annotation and quantification of alternative splicing events:** For each gene, all the reads with at least one base overlapping the gene from the start to the end coordinates are retrieved. CIGAR strings are then used to find the alignments blocks. Junction reads are identified by the presence of at least one’N’ letter in the CIGAR. Junction reads were filtered if:

- More than 10% of soft-clipping was detected in the alignment (it should not be the case with TopHat).
- An indel was close to the junction site, as it would make the junction position uncertain.

Junction read alignments are then processed block by block sequentially from left to right. Alignment blocks under 4 bp on read extremities are removed from the reads as we considered it is not sufficient to identify correctly the mapping localization. Then each block is compared to FasterDB annotations to check if the block boundaries correspond to known exons annotated in FasterDB, or to a putative new acceptor or donor site. First and last alignment blocks for each read must overlap one and only one exon for a read to be considered. For the inner blocks, if alignment blocks map to a succession of exons and introns, it is considered as an intron retention. For the acceptors and donors, we also added a supplementary filter. If a new donor is identified within a junction, we check if the junction also has an acceptor identified of the same length +/−1bp on the other side of the junction, showing most probably a problem of mapping. Once all the blocks are identified, the block annotations are used to annotate putative alternative splicing events: alternative skipped exon, multiple exon skipping, acceptor, or donor sites.

Once all the junction reads are processed, the alternative splicing events identified are pooled and the reads participating to each event are quantified, as well as the known exon-exon junction. If an exon-exon junction is annotated with multiple known acceptors and/or donors, all the possible junction reads are quantified and summed up. To fasten the quantification step, a junction coordinate file with the corresponding read numbers is produced from the read alignment using the same filters than described above and will be used for all the quantification tools: junction, exon skipping, acceptor and donor.

A challenge in defining the alternative skipped exon events is to identify the flanking exons. In the first version of FaRLine, these flankings exons were defined as the closest annotated genomic exons. This rule led to miss a lot of ASE events. Therefore, to define the flanking exons, we now use the information contained in the transcripts and in the reads. We consider each junction which skips an exon and is covered by at least one read. If this junction is annotated in the transcripts, we extract all annotated events containing this junction. Else, we annotate the event with the longest covered inclusion isoform. It allows FaRLine to be more robust to the incompleteness of the annotation compared to other methods, like MISO (Supplementary Figure S6). Panel C of Supplementary Figure S8 gives an example of an ASE reported by FaRLine but not by MISO because the exclusion isoform is not annotated in the transcripts.

**Comparison with STAR:** We also mapped the reads with STAR, ran FaRLine on these alignments and compared the predicted skipped exons with KisSplice. The main results are similar to what we found with TopHat. Indeed, without any filter, 69% of ASE annotated by KisSplice are also found by FaRLine and 24% of FaRLine’s event by KisSplice (compared to 68% and 24% respectively for the mapping with TopHat). When we filter out the events with an unfrequent variant, we show that approximately 70% of predicted ASE are found by both approaches.

#### Quantification and differential analysis

Both pipelines perform ASE detection and quantification. The quantification step was done similarly in the two pipelines where only the junction reads were taken into account. To evaluate if using exonic reads in the quantification could increase the accuracy of our methods, we ran KisSplice on the MCF-7 dataset with the option –exonic reads set to on. In doing so, only the inclusion rate of the AS events changes. When comparing usage of only junction reads to usage of both junction and exonic reads, we observed that the p-values calculated strongly correlate as shown in Supplementary Figure S16. We found that some AS events became significant upon the addition of exonic reads but the opposite also happened. Inspection of these events revealed that many are borderline cases, where the p-value is close, but slightly above 5%. A manual inspection of the AS events with a very different p-value upon addition of exonic reads revealed that they correspond to exons overlapping alternative first or last exons (see *STARD4*, Supplementary Figure S17A) or novel exons located in poorly spliced introns (see *PANK2* and *PRRC2B*, Supplementary Figure S17 B and C). Overall, we concluded that exonic reads can bring some statistical power in cases where the skipped exon does not overlap with any other event. In case of more complex events, exonic reads tend to “pollute” the pairwise comparison.

The last step of the pipelines is the differential analysis of the expression levels of the variants. This task is performed using the kissDE^24^ R package, which takes as input a table of read counts as in Supplementary Figure S18, and outputs a p-value and a DeltaPSI (Percent Spliced In).

Our statistical analysis adopted the framework of count regression with Negative Binomial distribution. We considered a 2-way design with interaction, with *isoforms* and *experimental conditions* as main effects. Following the Generalized Linear Model framework, the expected intensity of the signal was denoted by *λ*_*ijk*_ and was decomposed as:

1 $$\mathrm{log}\,{\lambda }_{ijk}=\mu +{\alpha }_{i}+{\beta }_{j}+{(\alpha \beta )}_{ij}$$

where *μ* is the local mean expression of the gene, *α*_*i*_ the contribution of splicing variant *i* on the expression, *β*_*j*_ the contribution of condition *j* to the total expression, and (*αβ*)_*ij*_ the interaction term. The target hypothesis was ${H}_{0}:\{{(\alpha \beta )}_{ij}=0\}$ *i.e*. no interaction between the variant and the condition. If this interaction term is not null, a differential usage of a variant across conditions occurred. The test was performed using a Likelihood Ratio Test with one degree of freedom. To account for multiple testing, p-values were adjusted with a 5% false discovery rate (FDR) following a Benjamini-Hochberg procedure^34^.

In addition to adjusted p-values, we report a measure of the magnitude of the effect. The measure we provide is based on the Percent Spliced In (PSI):

2 $$PS{I}_{condition}=\frac{count{s}_{{variant}1}}{count{s}_{{variant}1}+count{s}_{{variant}2}}$$

If counts for a variant are below a threshold, then the PSI is not calculated. This prevents from over-interpreting large magnitudes derived from low counts. When several replicates are available for a condition, then a PSI is computed for each replicate, and we calculate their mean.

Finally, we output the DeltaPSI:

3 $$DeltaPSI=PS{I}_{condition1}-PS{I}_{condition2}$$

unless one of the mean PSI of a condition could not be estimated. The higher the DeltaPSI, the stronger the effect. In practice, we consider only DeltaPSI larger than 0.1, a threshold below which it is difficult to perform any experimental validation.

### SK-N-SH dataset

We downloaded a total of 959 M reads from http://genome.crg.es/encode_RNA_dashboard/hg19/35. They correspond to long polyA+ RNAs generated by the Gingeras lab, and are also accessible with the following accession numbers (ENCSR000CPN - SRA: SRR315315, SRR315316 and ENCSR000CTT -SRA: SRR534309, SRR534310). For cell lines treated by retinoic acid, the reads were 76nt long, while they were 100nt long for the non treated cells. Hence we trimmed all reads to 76nt.

### MCF-7 dataset

MCF-7 were transfected (two biological replicates) with siRNA targeting both DDX5 and DDX17 RNA helicases, and total RNA were extracted as described previously^36^. cDNA synthesis was made using the TruSeq Stranded Total RNA protocol after Ribo-Zero Gold-mediated elimination of ribosomal RNA (Beckman Coulter Genomics). High throughput sequencing (2 × 125 bp) was carried out on an Illumina HiSeq 2500 platform (Beckman Coulter Genomics), generating between 45 and 50 millions of paired-end pairs of reads. Raw datasets are available on GEO under the accession number GSE94372.

Reads were trimmed according to standard quality control filters using prinseq^37^ and adapter were removed using cutadapt^38^. The resulting reads had length between 25 and 125nt. Because MISO is unable to deal with reads of unequal length, we selected only reads with length larger than 100nt (87% of the reads) and trimmed longer reads to 100nt.

### Computational requirements, software availability and reproducibility of the results

FaRLine took 45 hours and 10 Go of RAM. The time-limiting step was TopHat2, which took 41 hours, even parallelised on 8 cores. When STAR was tested instead of TopHat2, it took 4 hours, but 30 Go of RAM. KisSplice took 30 hours and 10 Go of RAM. The RAM-limiting step was STAR which took 30Go of RAM. All the steps of the pipelines can be reproduced using the following tutorial:

http://kissplice.prabi.fr/pipeline_ks_farline.

### Experimental Validation

SK-N-SH cells were purchased from the American Type Culture Collection (ATCC) and cultured using EMEM medium (ATCC) complemented with 10% FBS (Thermo Fisher Scientific). Cells were differentiated for 48 h using 6 μM of all-trans retinoic acid (Sigma-Aldrich).

After harvesting, total RNA were extracted using Tripure isolation reagent (Sigma-Aldrich), treated with DNase I (DNAfree, Ambion) for 30 min at 37 °C and reverse-transcribed (RT) using M-MLV reverse transcriptase and random primers (Invitrogen). Before PCR, all RT reaction mixtures were diluted at 2.5 ng *μ*L of initial RNA. PCR reactions were performed using GoTaq polymerase (Promega).

MCF7 cells were cultured as described in^36^. RT-PCRs were performed using the same protocol as for SK-N-SH cells.

## Electronic supplementary material

Supplementary Information

Supplementary Table 1

Supplementary Table 2
