# Supplementary material for: Complementarity of assembly-first and mapping-first approaches for alternative splicing annotation and differential analysis from RNAseq data
Source: Sci Rep. 2018 Mar 9;8:4307. doi: 10.1038/s41598-018-21770-7 (PMC5844962; doi:10.1038/s41598-018-21770-7)
Supplement: Supplementary file 1 — Supplementary Information [file 41598_2018_21770_MOESM1_ESM.pdf]

Complementarity of assembly-first and mapping-first approaches for alternative splicing annotation and differential analysis from RNAseq data

Clara Benoit-Pilven[1], Camille Marchet[3], Emilie Chautard[1,2], Leandro Lima[2], Marie-Pierre Lambert[1], Gustavo Sacomoto[2], Amandine Rey[1], Audric Cologne[2], Sophie Terrone[1], Louis Dulaurier[1], Jean-Baptiste Claude[1], Cyril F. Bourgeois[1], Didier Auboeuf[1,*], and Vincent Lacroix[2,*]

[1]Université de Lyon, ENS de Lyon, Université Claude Bernard, CNRS UMR 5239, INSERM U1210, Laboratory of Biology and Modelling of the Cell, 46 Allée d'Italie Site Jacques Monod, F-69007, Lyon, France

[2]Université de Lyon, F-69000, Lyon ; Université Lyon 1 ; CNRS, UMR5558, Laboratoire de Biométrie et Biologie Evolutive, F-69622, Villeurbanne, France. EPI ERABLE - Inria Grenoble - Rhône-Alpes

[3]IRISA Inria Rennes Bretagne Atlantique CNRS UMR 6074, Université Rennes 1, GenScale team, Rennes, 263 Avenue Général Leclerc, Rennes, France

[*]Corresponding authors : Vincent Lacroix (Vincent.lacroix@univ-lyon1.fr) and Didier Auboeuf (Didier.auboeuf@inserm.fr)

Supplementary methods :

KisSplice

Alternative splicing events are bubbles in the DBG

Supplementary figure S13 gives a schematic example of two alternative transcripts which differ by the inclusion of one exon. For the sake of simplicity, the example is given for words of length 3, but the reasoning holds for any word length. Each distinct word of length k is called a k-mer and corresponds to a node of the DBG. There is a directed edge from a node u to a node v if the last $k-1$ nucleotides of u are identical to the first $k-1$ nucleotides of v. Each transcript will therefore correspond to a path in the DBG. A pair of internally node-disjoint paths with a common source and target is called a bubble. The smaller path of the bubble corresponds to the exclusion isoform and is composed of all k-mers which overlap the junction between the exons flanking the skipped exon. It is therefore usually composed of $k-1$ k-mers. In the special case where the skipped exon shares a prefix with its 3' flanking exon, or a suffix with its 5' flanking exon, then the lower path is composed of less than $k-1$ k-mers and the k-mer which is the source (resp. target) does not correspond anymore to an exonic k-mer, but to a junction k-mer.

In practice, the DBG is built from the reads, not from the transcripts. The reads stem from possibly all genes expressed in the studied conditions.

Two difficulties arise: reads contain sequencing errors, and repeats may be shared across genes.

Dealing with sequencing errors

As originally described in[1] and later in[2], sequencing errors generate recognisable structures in De Bruijn graphs, which can be identified and removed. Their systematic removal however prevents assemblers from studying SNPs. A compromise consists in discarding rare k-mers from the graph. This is the strategy we use in KISSPLICE, where we remove all k-mers seen only once. This idea is however not sufficient in the context of transcriptome assembly, where the coverage is very uneven and mostly reflects expression levels. For highly expressed genes, several reads may have errors at the same site, generating k-mers with a coverage larger than an absolute threshold. We therefore also use a relative cut-off, which we set to 2%. These cut-offs we introduce to remove sequencing errors have an impact on the running time and on the sensitivity. Decreasing them allows to discover rarer isoforms, at the expense of a longer running time.

Dealing with repeats

Repeats are notoriously difficult to assemble in DNAseq data, and were initially thought to be much less problematic in RNAseq, since they are mostly located in introns and intergenic regions. In practice, mRNA extraction protocols are not perfect, and a fraction of pre-mRNA remains (typically 5% for total polyA+ RNA[3]). Each intron is covered by few reads, but if a repeat is present in many introns, then this repeat will obtain a high coverage. If, in addition, the multiple copies of the repeat are not identical, the repeat family will correspond to a very dense subgraph in the De Bruijn graph built from the reads. The traversal of such subgraph to enumerate all the bubbles it contains is long and mostly fruitless, although some true AS events flanked by

repeats may be trapped in these subgraphs. We showed in[4] that an effective strategy to deal with this issue is to enumerate only bubbles which have at most *b* branches. In practice, we set *b* to 5. Increasing *b* will increase the running time, but allow to find more repeat-associated alternative splicing events. Bubbles which do not correspond to true AS events can be filtered out at the mapping step.

MISO

MISO[5] was run in ”exon-centric” mode with default parameter. We first generated from the EnsEMBL r75 gff file the alternative event annotation file requested by MISO using `rnaseqlib`. The mapping step was done exactly the same as for FARLINE with Tophat-2.0.11[6], except that the replicates of each condition were merge together because MISO does not accept biological replicates. We then run all MISO scripts with default parameters. Finally, we filtered the differentially changing events with the `filter_events` script using the following parameters :
`--num-sum-inc-exc 10 --delta-psi 0.1 --bayes-factor 20`.

Cufflinks

Cufflinks[6] was run on the same alignment files used in FARLINE using annotation as a guide with the following parameters :
`-g <EnsEMBL r75 gff file> -b <hg19 genome> -u -p 16`.
When an annotation is given as a guide to Cufflinks, some faux-reads are introduced to support all transcripts present in the annotation. Because it can annotate transcripts even if there are not expressed in the samples, for the rest of the analysis, we decide to consider only the reconstructed transcripts supported by real reads.
Then, the AS events were retrieved from the reconstructed transcripts using the FARLINE annotation script.

Trinity

Trinity[7] was run with the following parameters :
`--max_memory 110G --CPU 16 --min_kmer_cov 2 --seqType fq --SS_lib_type RF`. In order to retrieve the bubbles from Trinity’s output file, we parsed the transcripts’ headers by firstly partitioning the reconstructed transcripts into disjoint sets, where each set is a predicted gene. Then, for each such set, the bubbles were found by processing the nodes’ identifiers used to build each isoform.

References

1. Pevzner, P. A., Tang, H. & Tesler, G. De novo repeat classification and fragment assembly. *Genome research* **14**, 1786–1796 (2004).

2. Zerbino, D. R. & Birney, E. Velvet: algorithms for de novo short read assembly using de bruijn graphs. *Genome research* **18**, 821–829 (2008).

3. Tilgner, H. *et al.* Deep sequencing of subcellular rna fractions shows splicing to be predominantly co-transcriptional in the human genome but inefficient for lncrnas. *Genome research* **22**, 1616–1625 (2012).

4. Sacomoto, G. *et al.* Navigating in a Sea of Repeats in RNA-seq without Drowning. *Lect.* **8701**, 82–96 (2014).

5. Katz, Y., Wang, E. T., Airoldi, E. M. & Burge, C. B. Analysis and design of rna sequencing experiments for identifying isoform regulation. *Nat. methods* **7**, 1009–1015 (2010).

6. Trapnell, C. *et al.* Differential gene and transcript expression analysis of rna-seq experiments with tophat and cufflinks. *Nat. protocols* **7**, 562–578 (2012).

7. Grabherr, M. G. *et al.* Trinity: reconstructing a full-length transcriptome without a genome from rna-seq data. *Nat. biotechnology* **29**, 644 (2011).

Supplementary figures :

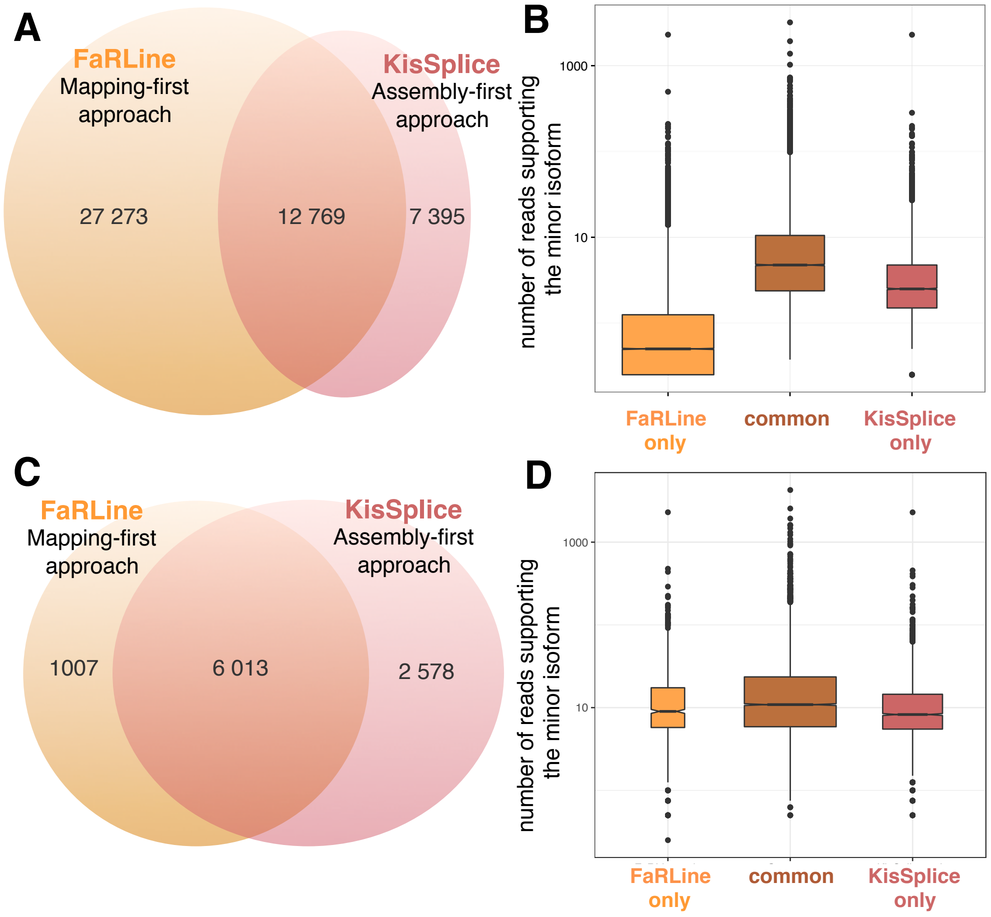

Figure S1. Comparison of the ASE identified by the assembly-first and mapping-first pipelines on MCF-7 dataset. A) Venn diagram of ASEs identified by the two pipelines. FARLINE detected many more events than KISSPLICE. 63% of ASE found by KISSPLICE were also found by FARLINE and 32% of ASE detected by FARLINE were also found by KISSPLICE. B) Boxplot of the expression of the minor isoform in the 3 categories defined in the Venn diagram of panel A: ASE identified only by FARLINE, ASE identified by both pipelines and ASE identified only by KISSPLICE. The number of reads supporting the minor isoform of the ASE identified by FARLINE is globally much lower. Many isoforms are supported by less than 5 reads. C) Venn diagram of ASEs found by the two pipelines after filtering out the poorly expressed isoforms. The common events represent a larger proportion than before filtering: 86% of the ASE annotated by FARLINE and 70% of the ASE annotated by KISSPLICE. D) Boxplot of the expression of the minor isoform in the 3 categories defined in the Venn diagram of panel C: ASE identified only by FARLINE, ASE identified by both pipelines and ASE identified only by KISSPLICE. The distribution of the number of reads supporting the minor isoform is similar for the 3 categories with highly expressed variants in each category.

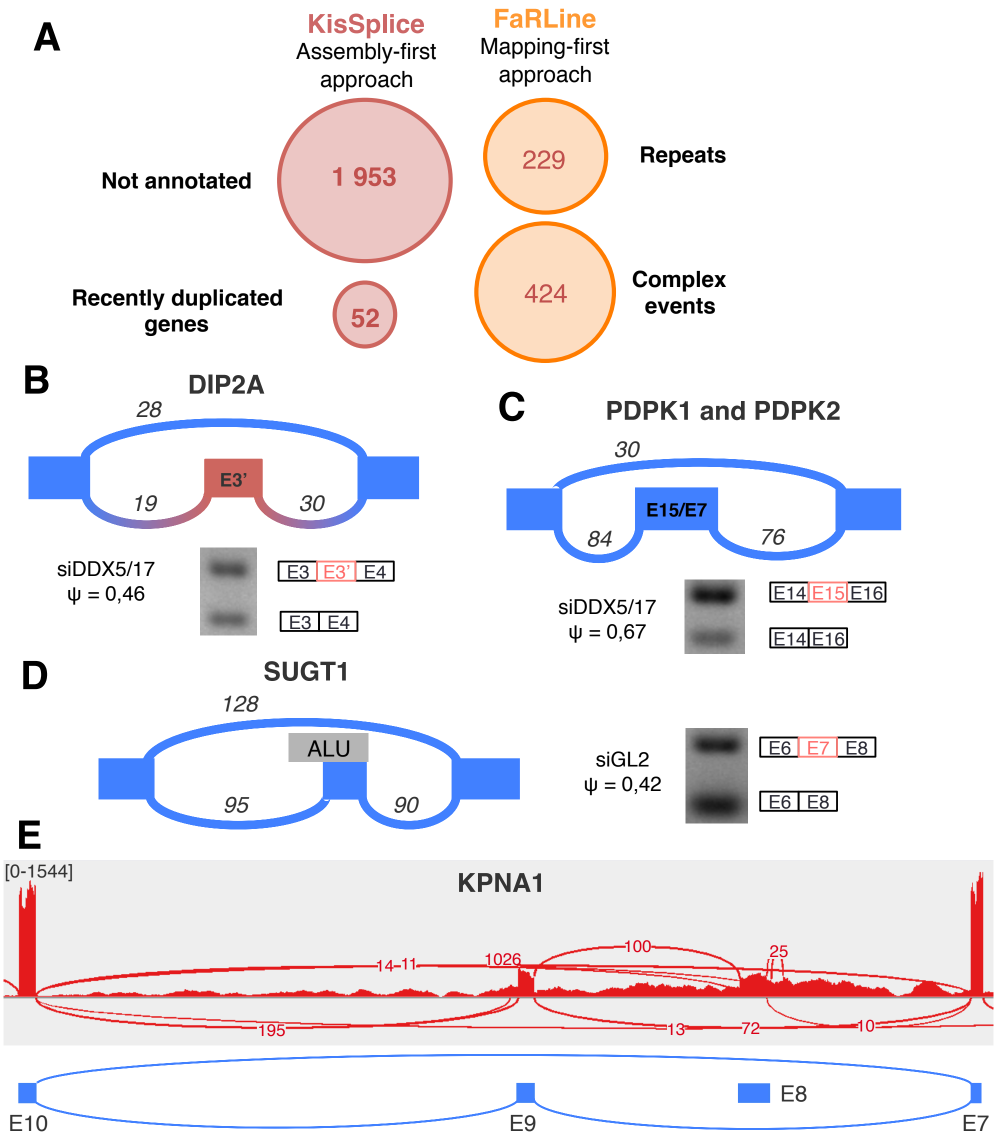

Figure S2. A) Main categories identified explaining why some exons are detected by only one method. Numbers for MCF-7 dataset. B) The exon in intron 3 of the *DIP2A* gene is an example of an exon not annotated in EnsEMBL r75. This event was identified by KisSplice but not by FaRLine. C) *PDPK1* and *PDPK2* are 2 paralog genes. KisSplice detected 2 isoforms that could be produced by these 2 genes. FaRLine did not detect any event in either of these genes. The exon skipped is exon 15 in *PDPK1* (corresponding to exon 7 in *PDPK2*). C) Exon 7 of the *SUGT1* gene is an example of exon skipping overlapping an Alu element identified only by FaRLine. The events in panel A to C were validated by RT-PCR. E) The *KPNA1* gene contains a complex event with more than 5 branches inside the bubble. This event was detected by FaRLinebut not by KisSplice

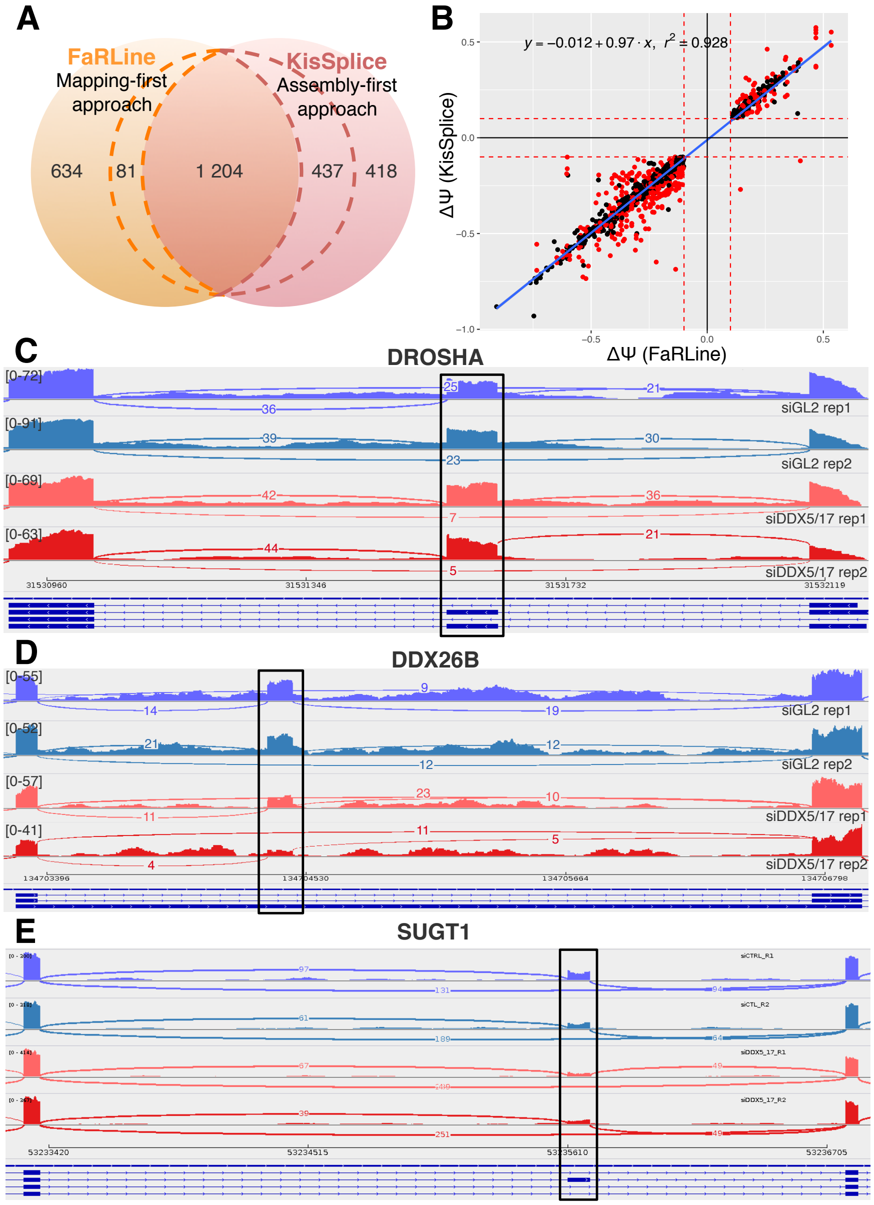

Figure S3. A) Condition-specific variants found by FARLINE, KISSPLICE or both methods in MCF-7 dataset. Within dashed lines are events identified by both approaches but detected as condition-specific by only one approach. B) DeltaPSI as estimated by KISSPLICE and FARLINE, for events identified by both methods. The red dots represent complex events for which KISSPLICE found at least 2 'bubbles'. C) Exon 2 of *DROSHA* is an example of regulated ASE found by both approaches. D) A new exon in intron 10 of the *DDX26B* gene is found only by KISSPLICE. The inclusion rate of this exon is differentially regulated between the 2 experimental conditions. E) Because exon 7 of the *SUGT1* gene is an exonised Alu element, only FARLINE identified this event. Moreover this exon is significantly more included in the control cells (expressing DDX5 and DDX17) when compared to the DDX5/DDX17 depleted cells.

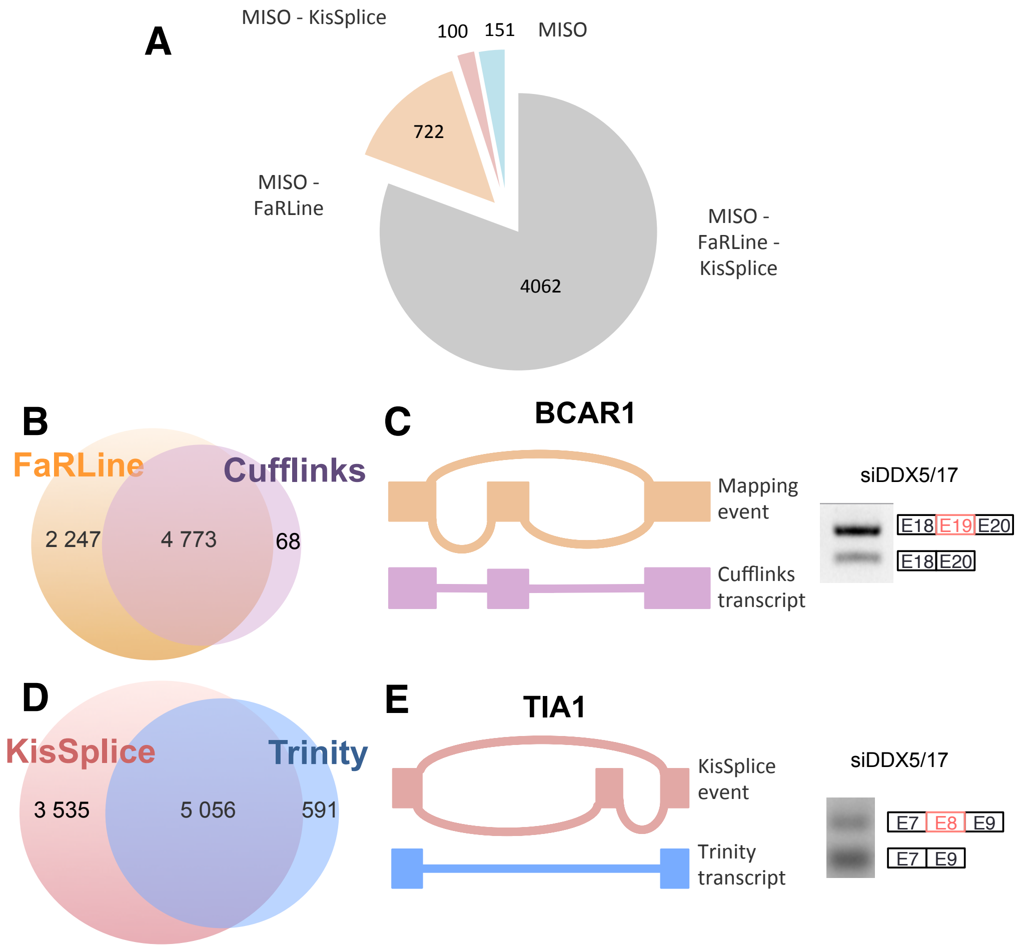

Figure S4. A) 81% of ASE found by MISO are also annotated by FARLINE and KISSPLICE. 14% of MISO's ASE are also annotated by FARLINE while only 2% of MISO's ASE are also annotated by KISSPLICE. Finally, only 3% of these ASEs are only annotated by MISO. B) Most of the events annotated by Cufflinks are found by FARLINE. C) *BCAR1* exon 19 is an example of an ASE annotated by FARLINE but not by Cufflinks. Indeed, only the inclusion isoform was identified by Cufflinks. D) Most of the events annotated by Trinity are also found by KISSPLICE. However half of the ASE annotated by KISSPLICE are not found by the global assembler Trinity. E) KISSPLICE annotates an ASE in the *TIA1* gene, while Trinity only identified the exclusion variant. The events in panels C and E have been validated by RT-PCR.

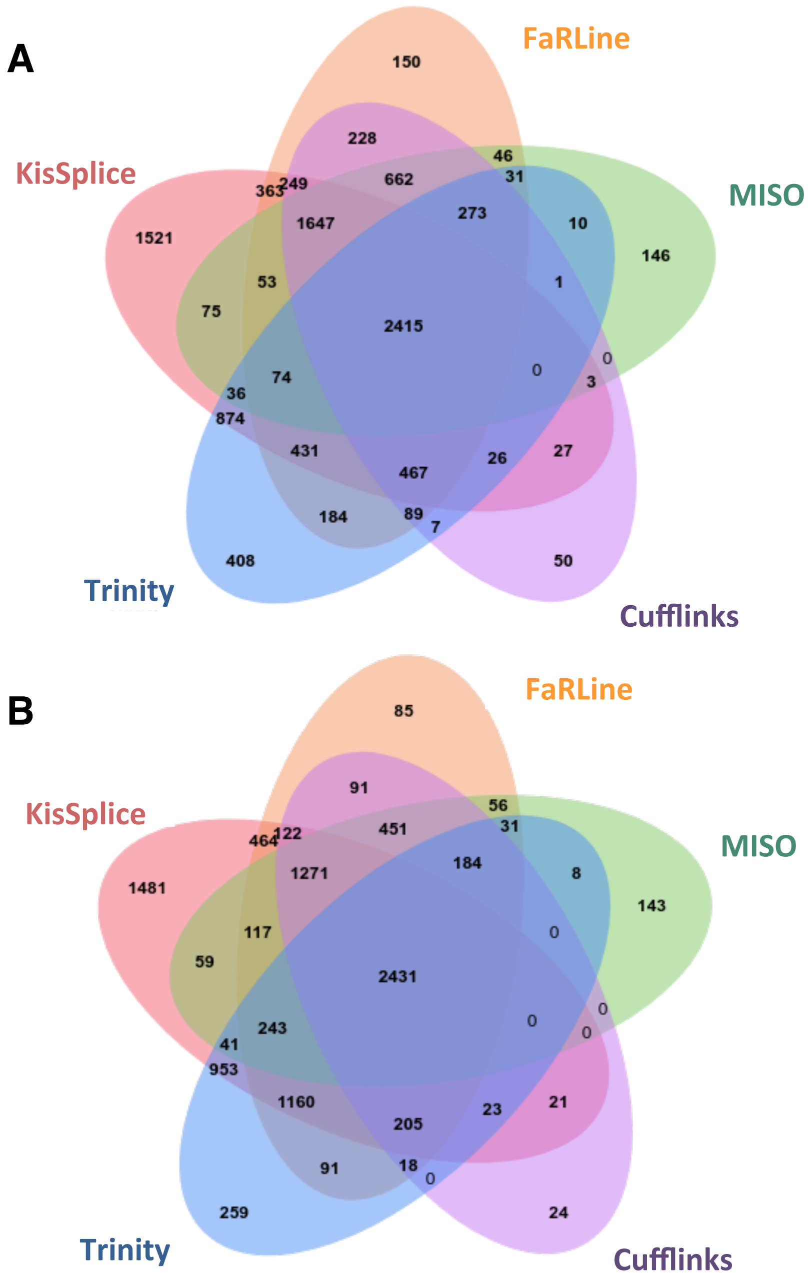

Figure S5. Venn diagram of the comparison of the five methods : KisSplice, FaRLine, MISO, Cufflinks and Trinity, on the SK-N-SH dataset (A) and on MCF-7 dataset (B). The total number of annotated splicing events predicted by at least one method, with the minor isoform being supported by at least 5 reads is 10546. The largest overlaps are 2415 (all methods), 1647 (all methods but Trinity), 874 (KisSplice-Trinity), 662 (FarLine-MISO-Cufflinks). As expected, Trinity is the least sensitive method. We also observe that the three mapping-first approaches (FarLine, MISO and Cufflinks) have a very large number of common candidates, 662 of which are not found by the two assembly-first approaches (KisSplice and Trinity). Conversely, the two assembly-first approaches have a very large number of common candidates, 874 of which are not found by the three mapping-first approaches. Similar numbers are found for the MCF-7 dataset. These results support the main conclusion of this paper.

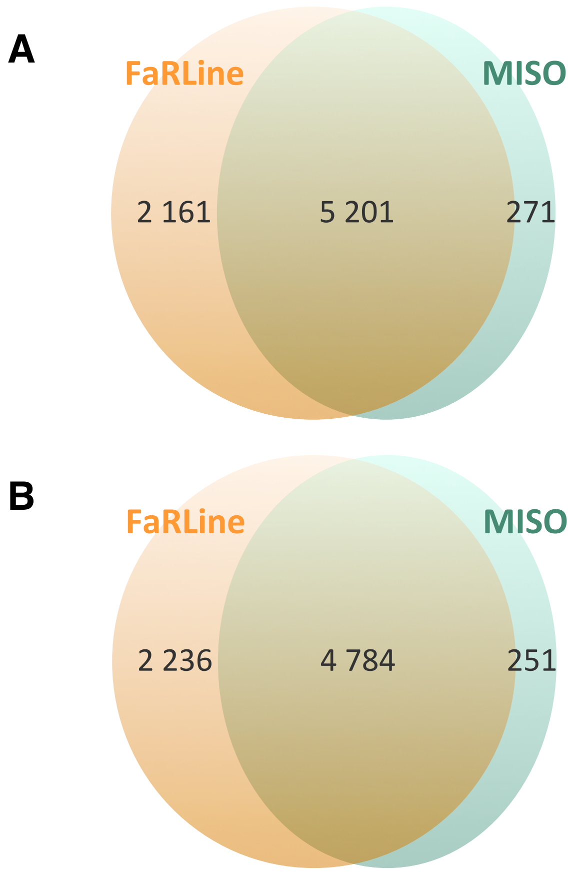

Figure S6. Venn diagram of the comparison of FaRLine and MISO on SK-N-SH dataset (A) and on MCF-7 dataset (B).

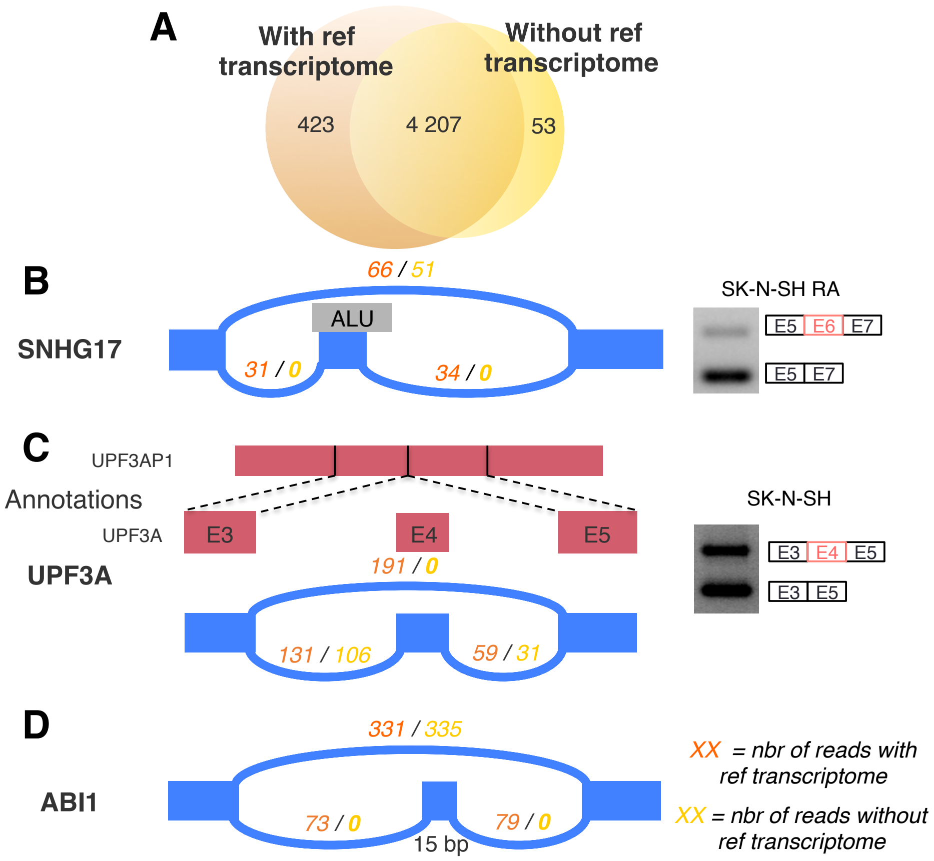

Figure S7. Comparison of the mapping-first approach FARLINE with or without an annotation provided to the mapper (i.e. with/without reference transcriptome) on the SK-N-SH dataset. A) More ASE are annotated when an annotation available. Panels B to D show examples of events only found by the mapping-first method when an annotation is provided to the mapper. B) The first category, represented by the *SNHG17* gene, includes exons containing repeats like ALU elements. C) Genes with a retrotransposed pseudogene, as *UPF3A*, represent the second category and are more difficult to find when no annotation is available. D) Short exons (less than 20 bp), like exon 5 of the *ABI1* gene, compose the third category.

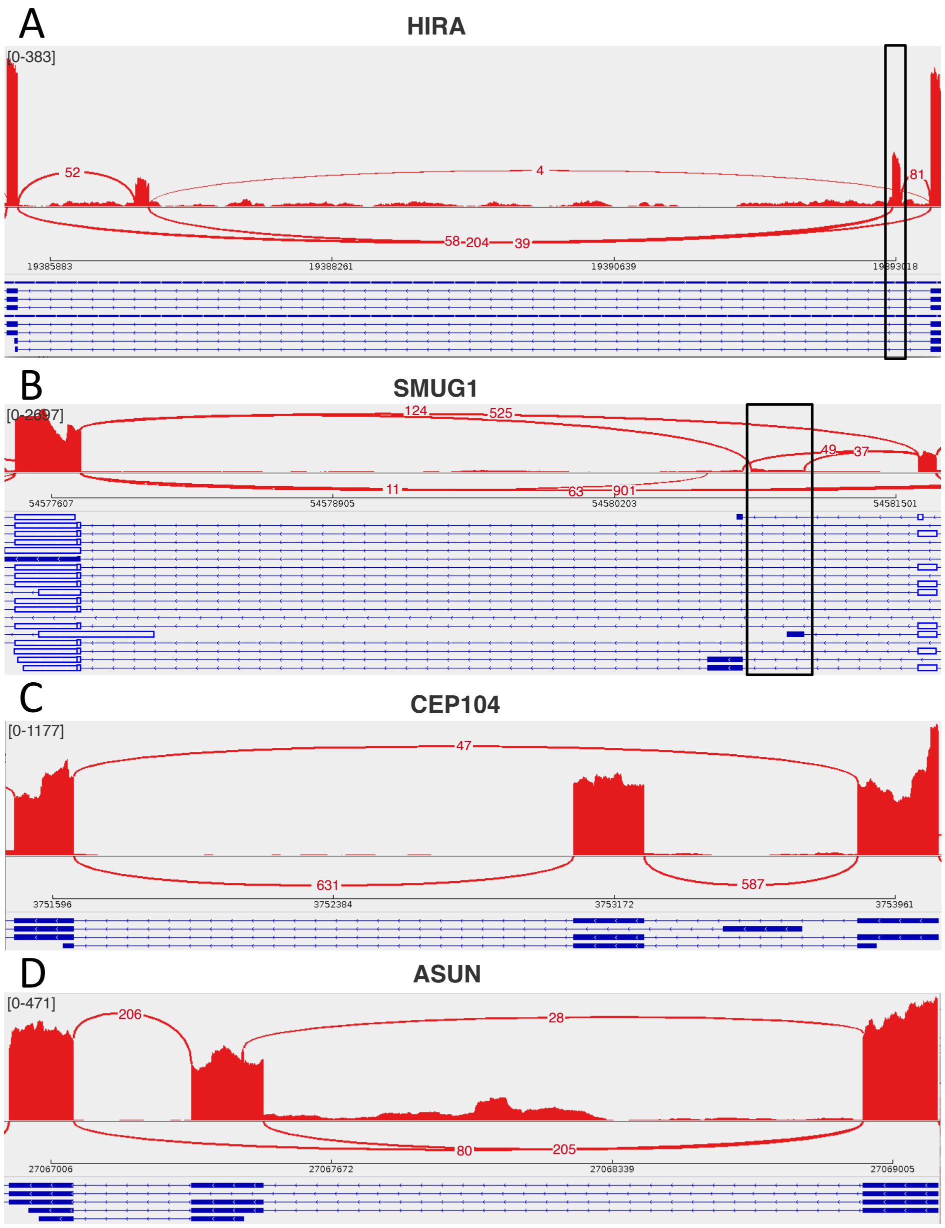

Figure S8. Examples of exon skipping inside a complex event. A) A new exon in intron 8 (black box) of *HIRA* gene is reported as skipped by KisSplice with exons 8 and 9 as flanking exons. B) The exon 5 of *SMUG1* gene is reported as skipped by KisSplice with exons 4 and 7 as flanking exons. This event is not found by FaRLine because the inclusion isoform is not annotated in the transcrits. C) Exon 12 of gene *CEP104* is reported as skipped by FaRLine even if the exclusion isoform is not present in the annotation. However, MISO does not find this exon skipping. D) Example of an exon skipping with two alternative donor sites in *ASUN* gene. It is reported as one event by FaRLine and two events by KisSplice.

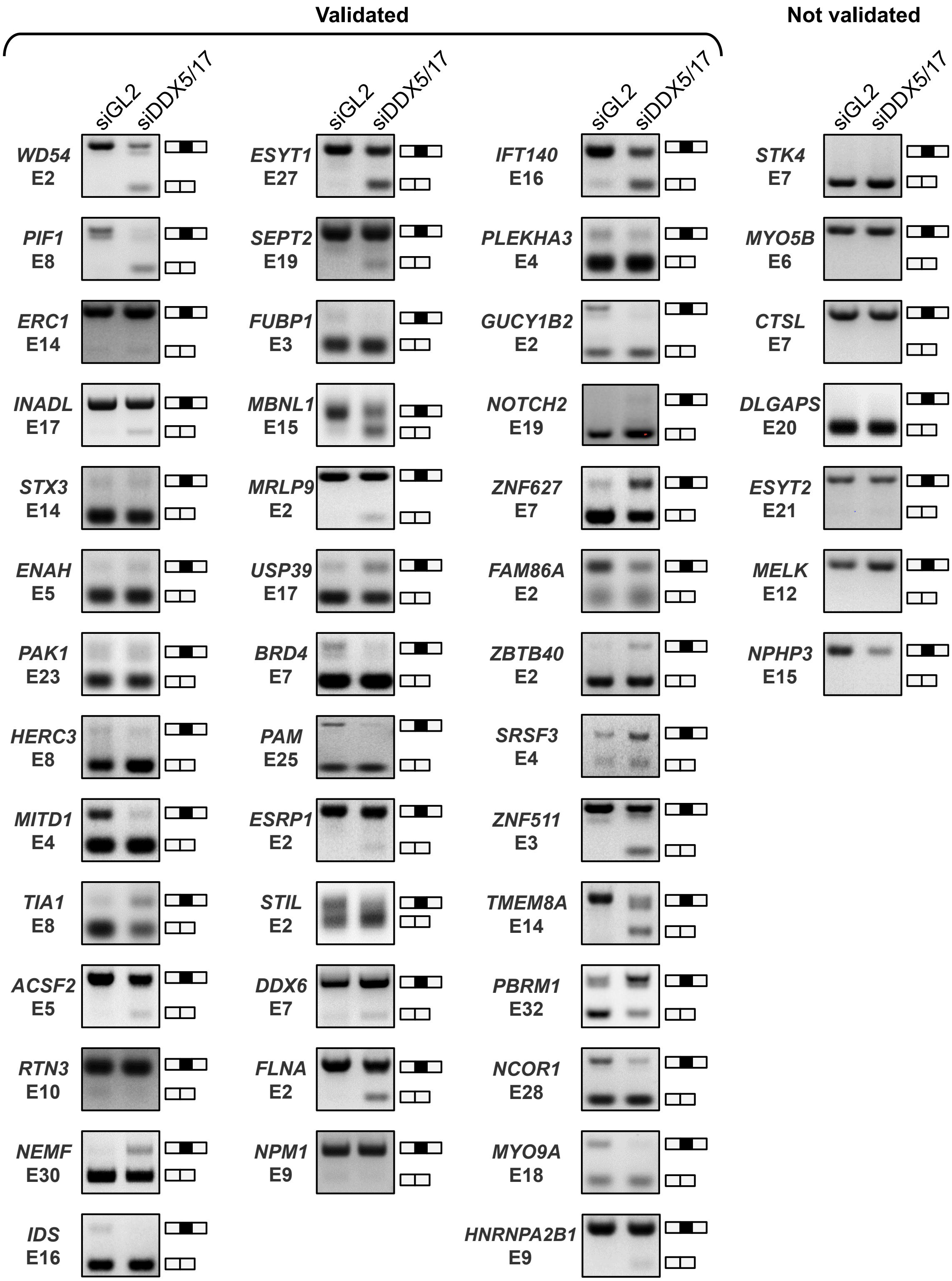

Figure S9. RT-PCR validations of events found by both approaches in the MCF-7 dataset. 41 out of the 48 events were validated (both the inclusion and the exclusion variant were amplified by RT-PCR). In some cases, there were additional PCR products (marked as '*') suggesting the existence of additional variants.

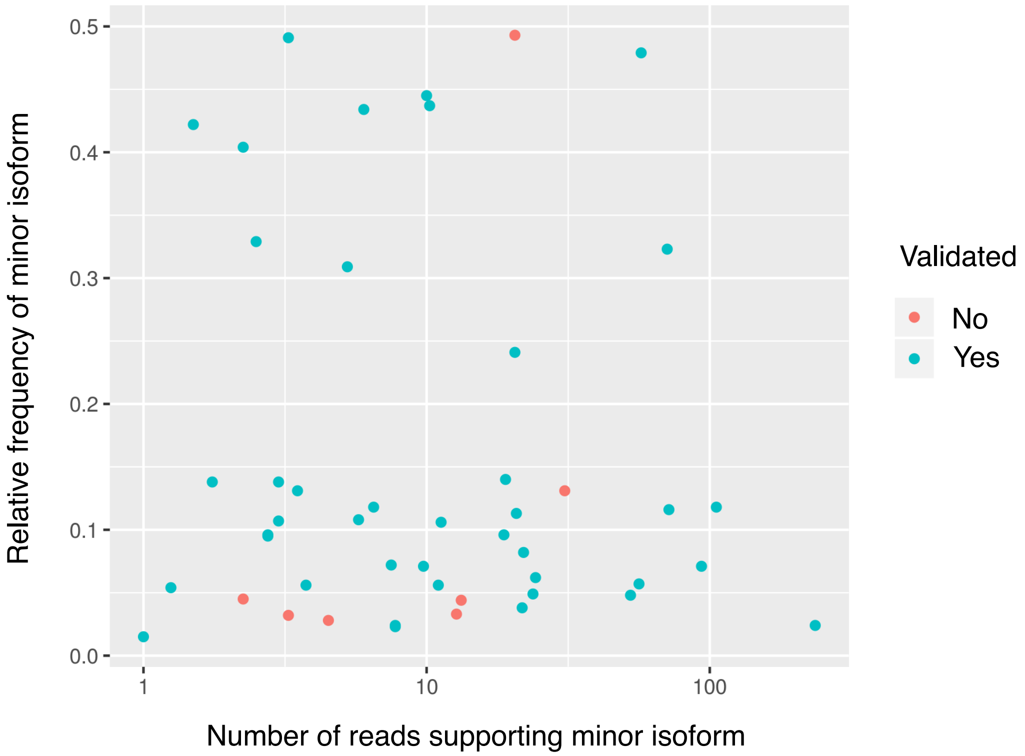

Figure S10. Repartition of validated and non validated ASEs according to number of reads supporting the minor isoform, and relative frequency of the minor isoform (i.e. number or reads of the minor isoform / number of reads supporting both isoforms). The X axis is in log scale. Most of the non validated cases have relative frequency of their minor isoform lower than 10%.

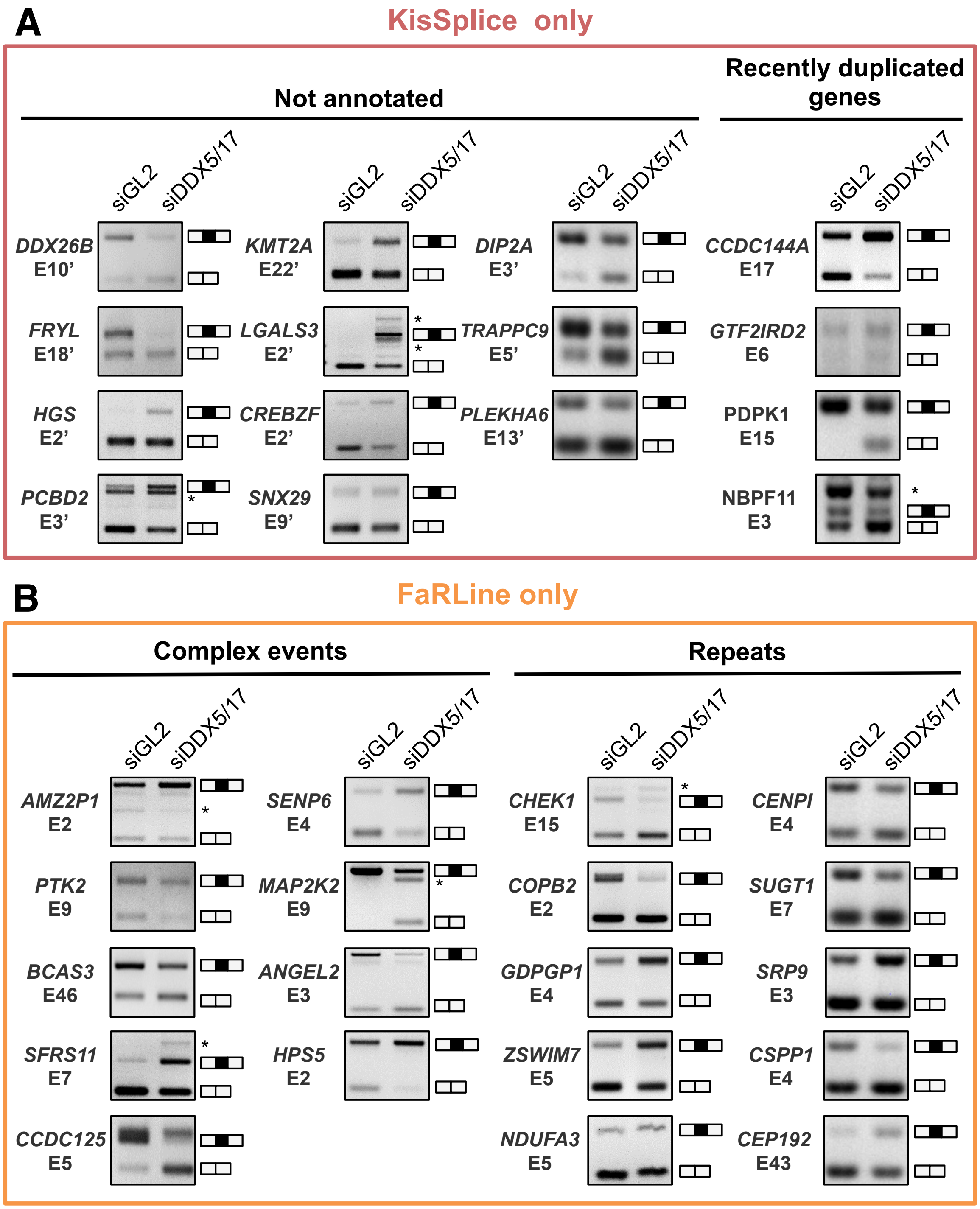

Figure S11. RT-PCR validations of events found only by KisSplice (A) and only by FaRLine (B) in the MCF-7 dataset. These ASEs were selected from the 4 main categories shown in Figure 3 and Supplementary Figure S2. All of them were validated.

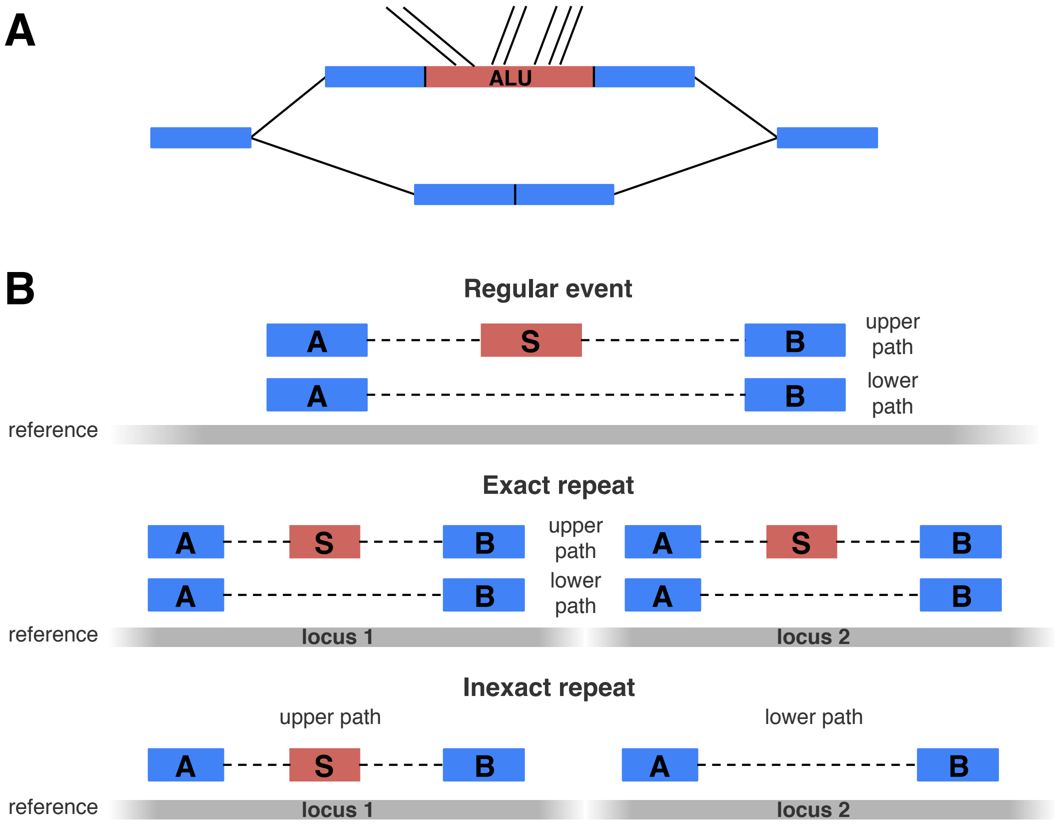

Figure S12. Dealing with repeats in KisSplice and KisSplice2RefGenome. A) Example of a bubble containing an Alu. Repeated events such as Alu are expected to be present in several copies in the reads. Thus, when the graph is constructed, edges link different copies of Alu. Because a bubble with more than 5 edges within one of its paths is not enumerated by KisSplice, this case is not annotated by the assembly-first approach. B) In KisSplice2RefGenome, if the two variants (i.e. paths) both map on different copies (exact repeat), we classify it as a recent paralog. On the contrary if each variant maps on a different locus, we consider the event as coming from an inexact repeat. This category represents mostly paralogs that have diverged.

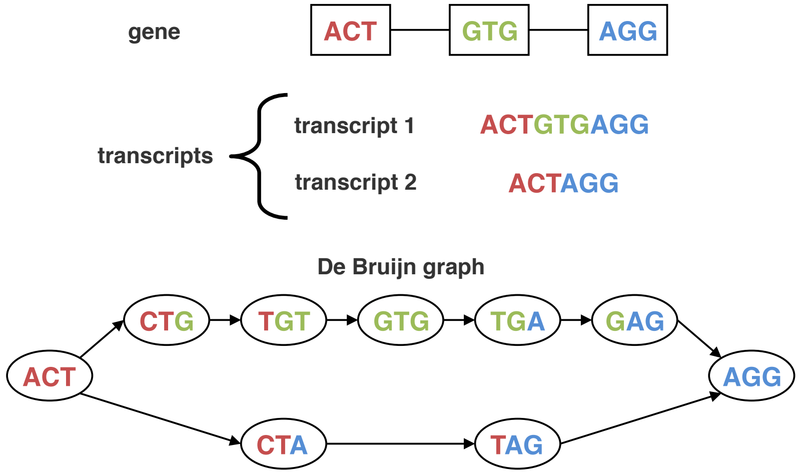

Figure S13. A schematic gene with three exons producing two alternative transcripts. The De Bruijn graph built from the sequences of the transcripts corresponds to a bubble. The upper path spells the skipped exon and its flanking junctions while the lower path spells the junction of the exclusion isoform and has a predictable length.

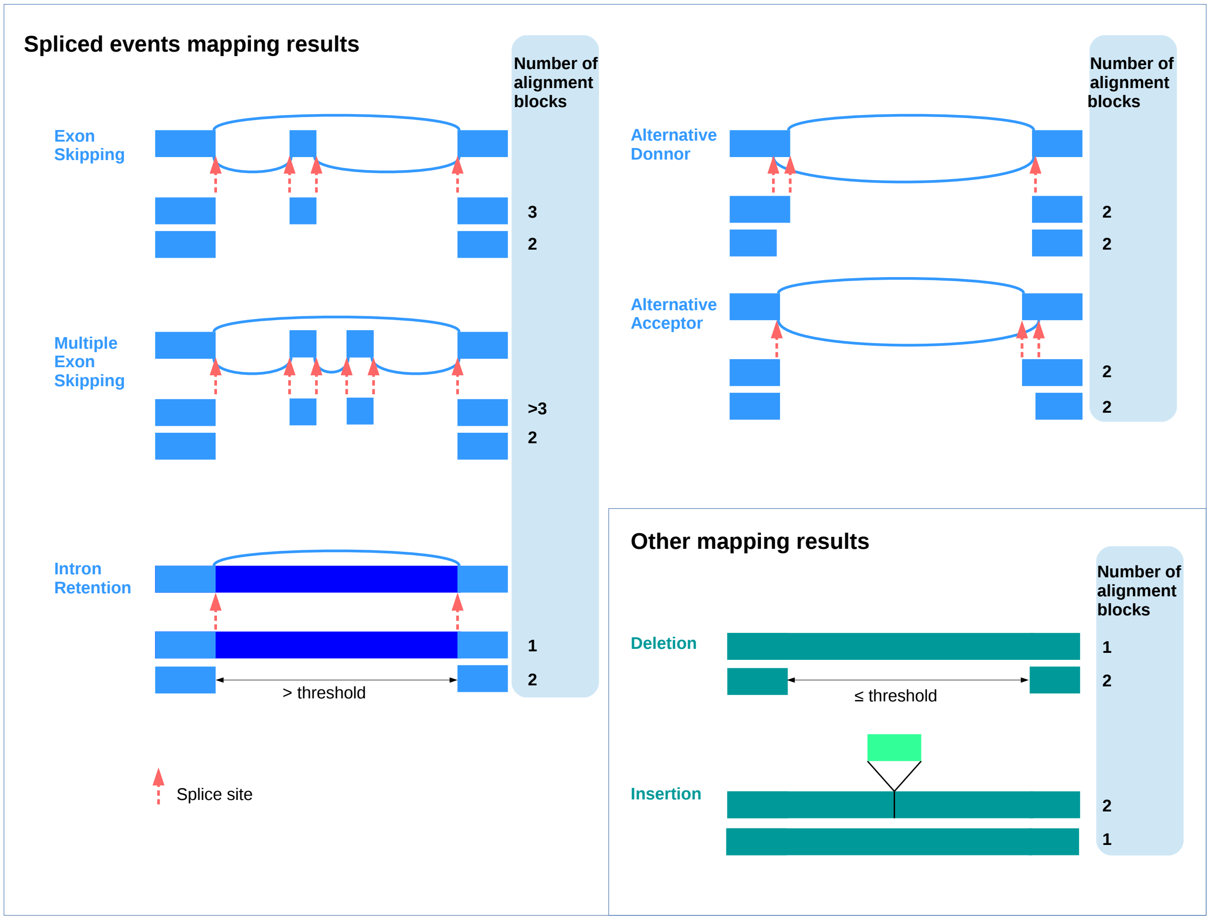

Figure S14. Classification of KisSplice events according to the number of blocks in which they map to the reference genome. Paths representing variants of an event are mapped on the reference. Spliced mapping results in blocks, events are then classified by KisSplice2RefGenome according to the block mapping patterns. (Putative) splice sites are noted by SS in red.

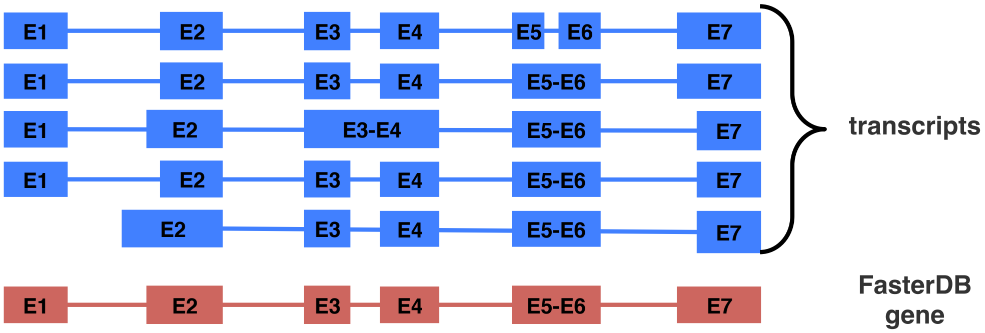

Figure S15. FasterDB exons are defined as the projection of the longer or most frequent exon in the transcripts (except for alternative first or last exons). The whole analysis done with FaRLine is based on these exons.

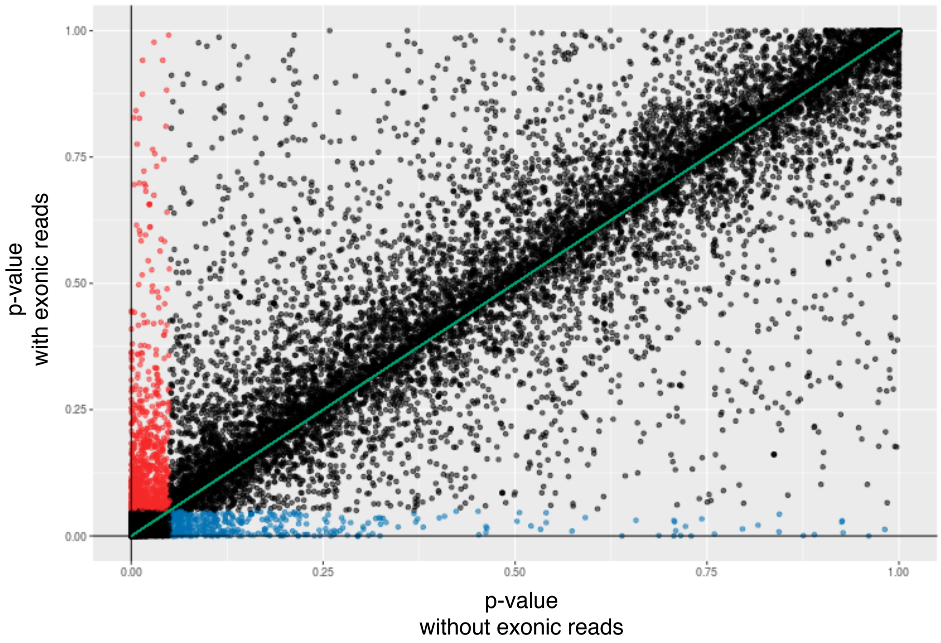

Figure S16. Correlation of the p-values when exonic reads were taken or not into account in the quantification. Red dots and blue dots correspond to ASE predicted to be regulated (p-value$<$0.05) when using junction reads and when using junction and exonic reads respectively.

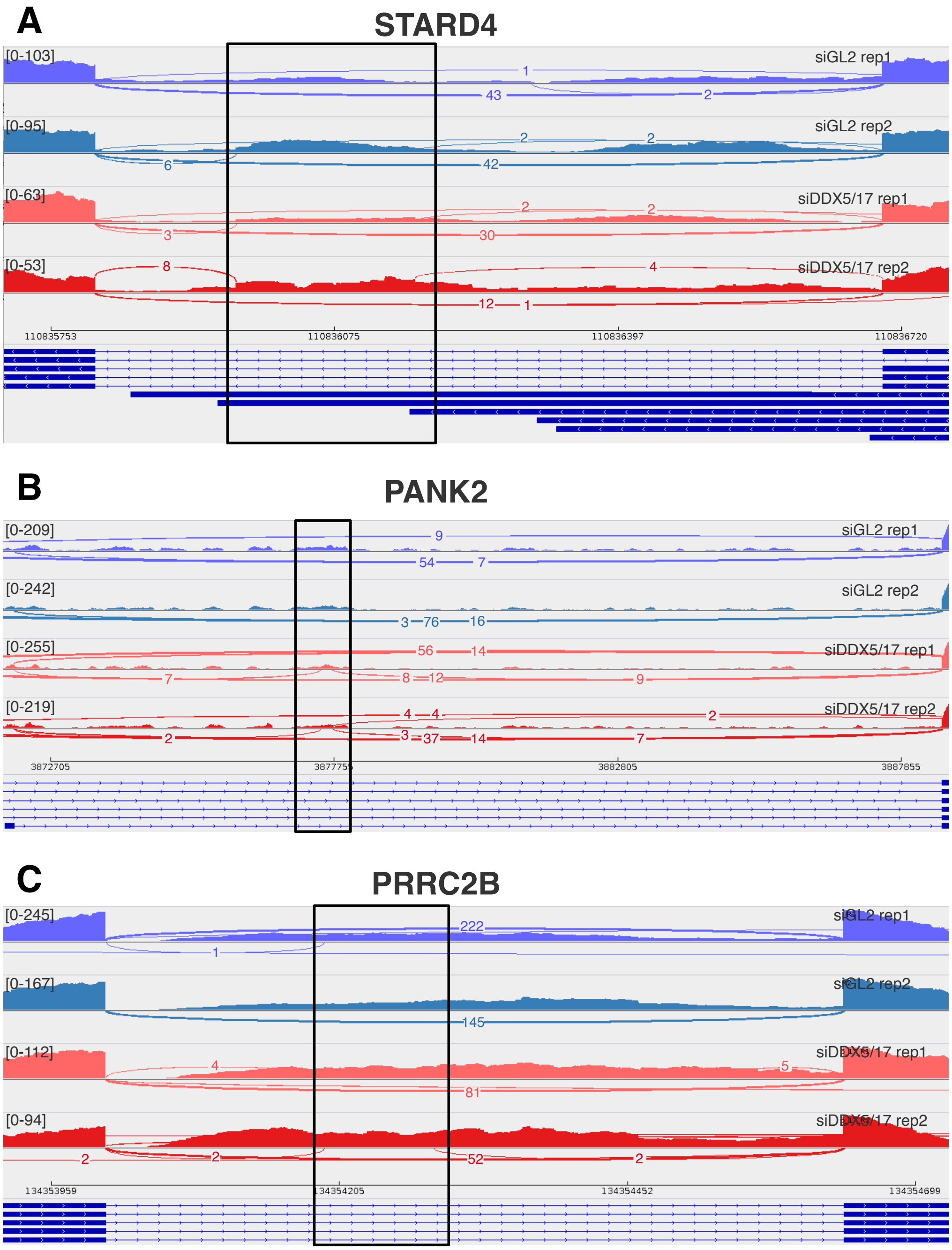

Figure S17. Examples of AS events predicted as differentially spliced between the two conditions in the MCF-7 dataset using junction and exonic reads, but not using only junction reads. A) Exon 6 of *STARD4* is detected as an alternatively skipped exon, but it also overlaps with an alternative last exon. B-C) Exon in intron 3 of *PANK2* gene and exon in intron 18 of *PRRC2B* gene are new exons found by KisSplice. These exons are located in poorly spliced introns.

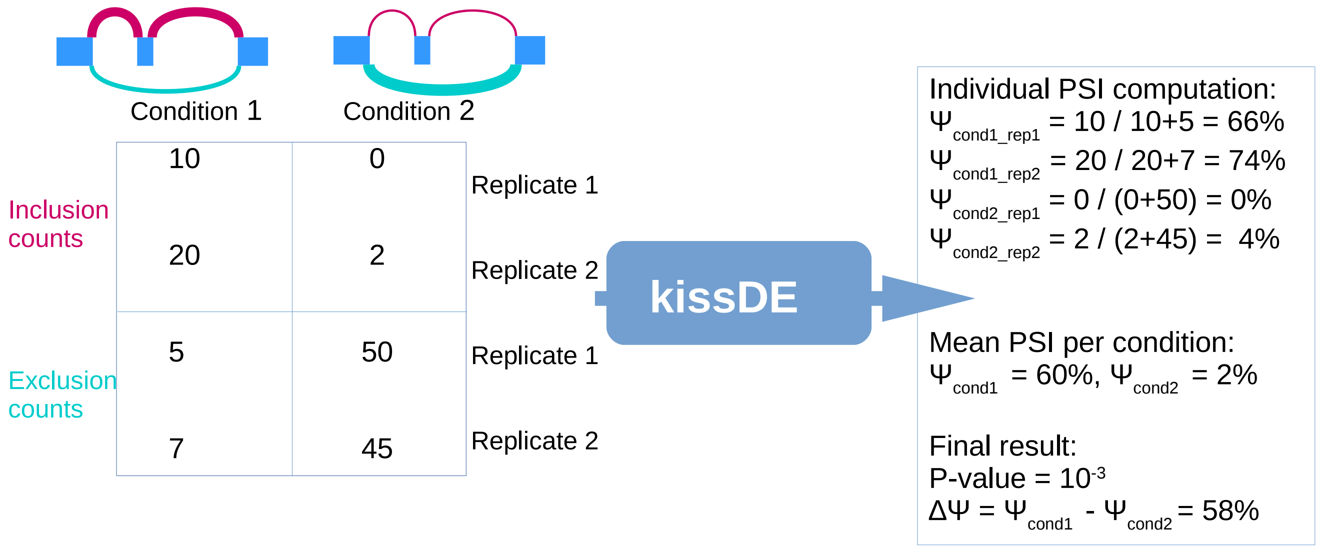

Figure S18. Input and output of the differential analysis. Counts for each replicate of each condition were computed by FaRLine or KisSplice. These counts together with the experimental plan are the input of kissDE. In this example, we show counts for one single event, in practice kissDE tests all events discovered by one method to spot the differential splicing events. Provided that at least two replicates are available per condition, kissDE computes p-values and DeltaPSI ($\Delta\Psi$) per event, and results are ranked using these two metrics.

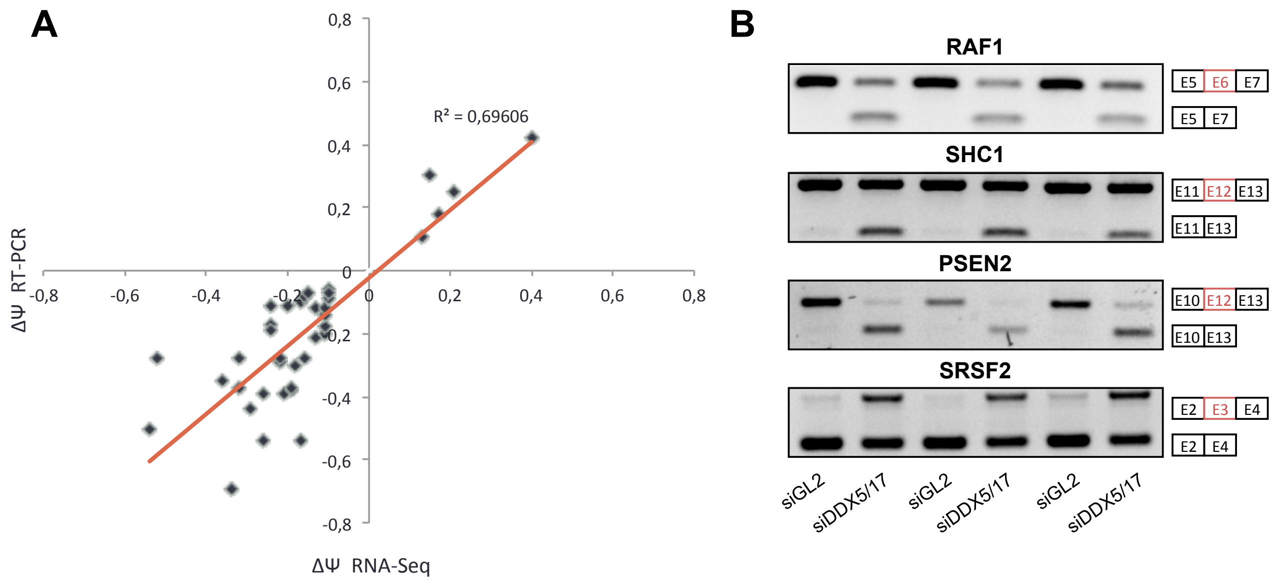

Figure S19. Validations of ASE regulated by the depletion of DDX5 and DDX17 in MCF7 cell line. A) Correlation of the deltaPSI computed from the RNAseq and the deltaPSI computed from the validations by RT-PCR. B) RT-PCR validations of some of the events regulated by the depletion of DDX5 and DDX17 in MCF7 cell line.
